# In Situ Engineered “Cascade‐Amplified” Drug‐Loaded Vesicles for Enhanced Cancer Stem Cell Therapy

**DOI:** 10.1002/jev2.70292

**Published:** 2026-05-09

**Authors:** Tiantian Zhang, YuanYuan Wei, Zimai Liu, Zixian Wu, Xiaoxi Wang, Kai Li, Hui Liu, Jiao Lu, Qianxi Lu, Meiyi Liu, Yongchao Wang, Zhenzhen Chen

**Affiliations:** ^1^ School of Life Sciences Zhengzhou University Zhengzhou Henan China; ^2^ School of Medicine Shihezi University Shihezi China; ^3^ School of Materials Science and Engineering Zhengzhou University Zhengzhou Henan China; ^4^ Longhu Laboratory of Advanced Immunology Zhengzhou China; ^5^ Institute of Aging Decoding and Regeneration, Zhengzhou University Zhengzhou Henan China; ^6^ The First Affiliated Hospital of Henan University Kaifeng China

**Keywords:** cancer stem cells, deep delivery, extracellular vesicles, immunotherapy

## Abstract

Cancer stem cells (CSCs) characterized by the capacity of self‐renewal and drug resistance, are a major cause of tumour recurrence and metastasis. However, CSCs are mainly localized in the deep and hypoxic regions of the tumour microenvironment that hinder drug penetration. Furthermore, their overexpression of the CD24/Siglec10 immune checkpoint axis markedly suppresses immune clearance, severely limiting the efficacy of current therapeutic strategies. To address this challenge, this study developed an in situ engineered “cascade‐amplified” drug‐loaded vesicle delivery system, aiming to achieve deep drug delivery into CSC‐enriched regions and enhance anti‐tumour immune responses. Based on a biomimetic “core‐shell” nanoplatform (siXkr8/Dox@PMLC), this system initiates a cascade within the TME where Doxorubicin (Dox) induces tumour cells to generate drug‐loaded apoptotic bodies (ApoBDs). These ApoBDs serve as primary vesicles that, upon uptake by adjacent tumour cells, trigger secondary apoptosis, establishing a “cascade‐amplified” cycle of enhanced drug delivery. Meanwhile, the silencing of the phospholipid scramblase Xkr8 via siRNA inhibits phosphatidylserine (PS) exposure on the surface of ApoBDs, thereby preventing their recognition and clearance by M2‐type macrophages and facilitating immune phenotype remodelling. Furthermore, through targeted blockade of the CD24/Siglec‐10 immune axis, the nanoplatform enhances macrophage‐mediated phagocytosis of CSCs. In summary, this strategy achieves deep eradication of CSCs and synergistically enhances anti‐tumour immunotherapy, demonstrating significant translational potential.

## 1 Introduction

Despite major advances in cancer treatment in recent decades, tumour recurrence and metastasis mediated by cancer stem cells (CSCs) remain a major barrier to durable clinical responses (Bayik and Lathia 2021; Jiang et al. 2025, Dakal et al. 2020; Liu et al. 2025). CSCs preferentially localize to hypoxic niches (Najafi et al. 2020; Koch et al. 2010), where they are shielded by a dense extracellular matrix, elevated interstitial fluid pressure, and disorganized vasculature—all of which severely impede drug penetration (Primeau et al. 2005; Minchinton and Tannock 2006). Moreover, CSCs overexpress the immune checkpoint molecule CD24 (KE et al. 2012; Salaria et al. 2015; Xu et al. 2024; Hu et al. 2024), which binds to the macrophage receptor Siglec‐10 to evade phagocytic recognition and clearance (Barkal et al. 2019). This immune evasion mechanism allows CSCs to persist in the tumour microenvironment, rendering them resistant to conventional therapies. Thus, developing drug delivery systems that can simultaneously target CSC‐enriched tumour regions and modulate host immunity to eliminate CSCs is a critical strategy for improving cancer treatment efficacy.

Extracellular vesicles (EVs) are membrane‐bound nanostructures naturally secreted by cells, playing pivotal roles in intercellular communication and biomolecule transport (Yáñez‐Mó et al. 2015). Owing to their exceptional biocompatibility, low immunogenicity, and intrinsic targeting properties, EVs have emerged as promising candidates for next‐generation drug delivery platforms (Herrmann et al. 2021; Wang et al. 2025; Lu et al. 2023; Ai et al. 2024). However, several critical challenges hinder their clinical translation, including complex isolation procedures, limited production yields, and inefficient drug loading capacity. Moreover, native EVs often require post‐isolation modifications for therapeutic cargo encapsulation or surface engineering (Li et al. 2025; Liang et al. 2025). Current standard loading techniques, such as extrusion (Su et al. 2020), sonication (Du et al. 2021), and solvent‐mediated methods (Haney et al. 2019), frequently compromise EVs’ membrane integrity and biological activity despite enhancing payload capacity, ultimately impairing their structural stability and in vivo delivery efficiency (Guo et al. 2023; Ma et al. 2025). Notably, various organisms (e.g., eukaryotic cells and bacteria) can autonomously secrete EVs in vivo, serving as natural carriers for biomolecular cargo and intercellular signalling (Yue et al. 2022; Kong et al. 2022). Therefore, utilizing endogenously generated EVs as drug or bio‐signal carriers may circumvent the cumbersome processes and stability issues associated with exogenously prepared EVs, offering novel strategies for drug delivery (Zhang et al. 2025).

Apoptotic bodies (ApoBDs), a subtype of endogenous EVs released during tumour cell apoptosis, exhibit potential as both natural intercellular messengers and drug delivery vehicles (Zhou et al. 2022). ApoBDs demonstrate superior biocompatibility and remarkable adaptability to the tumour microenvironment (TME) (Yu et al. 2023). Their native cell‐derived membrane structures enable selective fusion or phagocytosis by tumour cells and CSCs, significantly enhancing cargo accumulation in CSC‐enriched niches. However, ApoBD‐mediated delivery faces critical limitations. During chemotherapy‐induced apoptosis, scramblases such as Xkr8 are activated, mediating the irreversible externalization of phosphatidylserine (PS) from the inner to the outer leaflet of the cancer cell membrane (Chen et al. 2023; Nagata et al. 2016). This results in abundant PS exposure on the surface of ApoBDs. By presenting “eat me” signals to macrophages, this process facilitates the recognition and clearance of ApoBDs (Lemke 2019), thereby severely limiting effective drug accumulation in tumour cells, particularly in CSCs. Furthermore, ApoBDs can polarize macrophages toward an immunosuppressive M2‐like phenotype by inducing secretion of cytokines (Zhang et al. 2019), thereby exacerbating tumour immune evasion. These challenges emphasize the potential of in situ ApoBD engineering as a strategy to concurrently improve CSC‐targeted drug delivery and enhance immune‐mediated tumour recognition and clearance.

**FIGURE 1:**
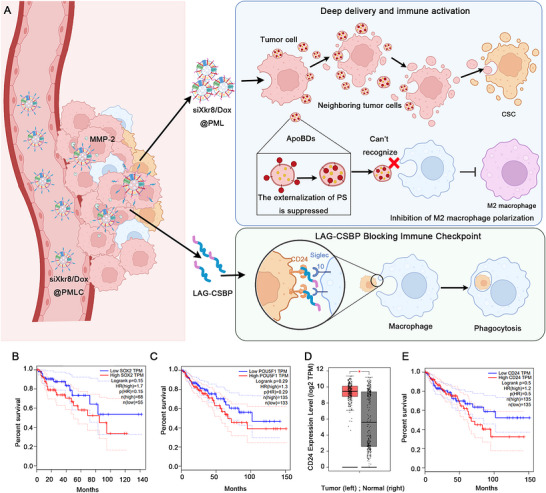
(A) Schematic of cancer stem cells clearance by the siXkr8/Dox@PMLC system. siXkr8/Dox@PMLC induces tumour cells to generate primary drug‐loaded ApoBDs in situ, which are then transferred to adjacent tumour cells to trigger secondary apoptosis. This forms a cascade‐amplified drug delivery system, thereby improving the accessibility of drug to CSCs. Meanwhile, the reduced exposure of PS on the surface of primary ApoBDs enables them to evade recognition and clearance by macrophages, inhibits macrophage polarization toward the M2 phenotype, and further enhances drug delivery efficiency. Additionally, the system blocks the CD24/Siglec‐10 signalling axis, which strengthens the phagocytic clearance efficiency of macrophages against CSCs. (B, C) Correlation between the expression of Sox2 (B), Oct4 (C) and overall survival in patients with colorectal cancer. (D) The expression levels of CD24 in colorectal cancer tissues and normal tissues. (E) Correlation between the expression of CD24 and overall survival in patients.

To address these challenges, we developed an in‐situ drug‐loaded ApoBD delivery system designed for cascade‐amplified therapeutic efficacy. This system employs a biomimetic core‐shell nanoplatform (siXkr8/Dox@PMLC) that synergistically induces continuous production and in situ modification of drug‐loaded ApoBDs in the TME, establishing a cascade‐amplified delivery process. The strategy achieves deep‐tissue drug penetration while continuously generating new drug‐carrying ApoBDs through cascading apoptosis, significantly enhancing both CSC‐targeted delivery efficiency and immune‐mediated clearance. The integrated platform comprises three functional modules (Figure 1A): (1) Dox serves as the initiator to induce primary tumour cell apoptosis and release drug‐loaded ApoBDs, which subsequently trigger secondary apoptosis in neighbouring tumour cells, creating a self‐sustaining ApoBD generation‐diffusion cycle; (2) Xkr8‐targeting siRNA suppresses PS exposure on ApoBDs to evade macrophage clearance while inhibiting M2 polarization; (3) MMP‐2‐responsive CD24‐blocking peptides (M‐CSBP) conjugated to the nanoplatform disrupt the CD24/Siglec‐10 immune checkpoint axis to enhance macrophage‐mediated CSC recognition and phagocytosis.

In summary, this study presents a cascade‐amplified strategy utilizing drug‐loaded ApoBDs through in situ tumour engineering. By regulating the generation and modification of ApoBDs, blocking immune evasion signals, and synergistically enhancing macrophage activity, this approach shows significant therapeutic potential.

## 2 Results and Discussion

### 2.1 CSCs Drive Therapy Resistance Through CD24‐Mediated Immune Evasion and Niche Barriers

Given the critical role of CSCs in tumorigenesis and progression, we first analyzed the association between colorectal cancer stemness‐related genes and patient prognosis using database mining. The results showed that high expression of stemness genes was significantly correlated with poor patient prognosis (Figure 1B–C), suggesting that CSCs may be a key factor contributing to therapeutic resistance in cancer. Further investigations revealed that CD24, a surface marker of CSCs, can promote tumour progression by mediating immune evasion mechanisms. Database analysis demonstrated that the expression level of CD24 in colorectal cancer tissues was significantly higher than that in normal tissues (Figure 1D). Moreover, patients with high CD24 expression showed a markedly reduced survival rate compared to those with low CD24 expression (Figure 1E). These findings indicate that CD24 is crucial for maintaining the survival of CSCs. Targeting and inhibiting CD24 may block its signalling crosstalk with Siglec‐10, thereby improving tumour treatment efficacy. In addition, studies have confirmed that CSCs are often located in deep tumour tissues far from blood vessels, where drug permeability is poor, enabling CSCs to evade drug‐induced killing and survive.

These findings indicate that the unique CSC niche and CD24 overexpression promote CSC maintenance. In this context, these discoveries reveal that simultaneously addressing the issue of drug accessibility to deep‐seated CSCs and the immune evasion mediated by the CD24/Siglec‐10 signalling axis is key to breaking through the current therapeutic bottleneck.

### 2.2 Characterization of siXkr8/Dox@PMLC

**FIGURE 2:**
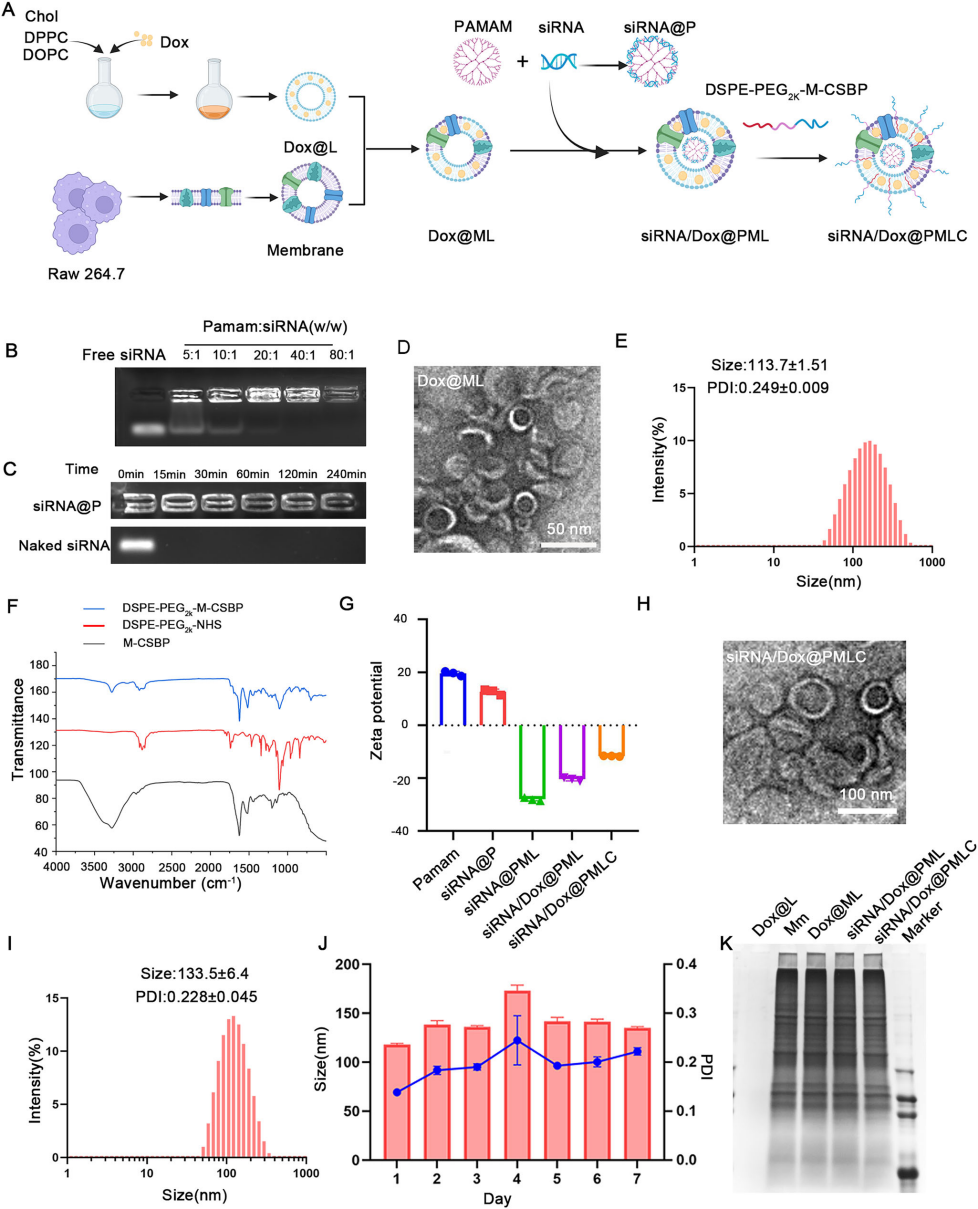
**Characterization of siRNA/Dox@PMLC** (A) Synthesis schematic of siRNA/Dox@PML. (B) Agarose gel electrophoresis of siRNA@P complexes prepared at various mass ratios. (C) Protection of siRNA from RNase A degradation. (D) TEM and (E) DLS characterization of Dox@ML. Scale bar: 50 nm. (F) Fourier‐transform ifrared spectroscopy (FTIR) mapping of DSPE‐PEG_2k_‐NHS conjugated with M‐CSBP. (G) Zeta potential changes during the preparation of siRNA/Dox@PMLC. (H, I) TEM and DLS characterization of siRNA/Dox@PMLC. Scale bar: 100 nm. (J) Stability of siRNA/Dox@PMLC in PBS, *n* = 3. (K) Sodium dodecyl sulfate‐polyacrylamide gel electrophoresis (SDS‐PAGE) analysis of membrane‐associated proteins.

The synthesis process of siXkr8/Dox@PMLC is shown in Figure 2A. Amino‐terminated polyamidoamine dendrimer (PAMAM), a cationic polymer with good biocompatibility, was used for loading siRNA (Chen et al. 2022; Biswas et al. 2013). siRNA@P was shown to have a homogeneous spherical structure with a size similar to that of PAMAM (≈10 nm) (Figure S1A and S1B). The siRNA loading capacity of PAMAM was verified by agarose gel electrophoresis across mass ratios (PAMAM: siRNA) ranging from 5:1 to 80:1. The results showed that when the mass ratio of PAMAM to siRNA was 40:1, the siRNA was completely bound by PAMAM and retained in the sample wells without free migration (Figure 2B). Furthermore, the RNase‐protective capacity of PAMAM in stabilizing siRNA was evaluated. Compared to naked siRNA, siRNA encapsulated in PAMAM retained its structural integrity (Figure 2C).

To enhance the biocompatibility and targeting capability of siRNA@P, we performed surface modification. Specifically, doxorubicin (Dox)‐encapsulating liposomes were first prepared by thin‐film hydration. The optimal encapsulation efficiency (79.8 ± 0.15%) and loading capacity (1.91 ± 0.04%) were achieved at a lipid‐to‐Dox ratio of 40:1 (w/w). These Dox@L were subsequently fused with extracted macrophage membranes (Mm) by co‐extrusion. To verify the fusion effect, we performed co‐localization experiments using confocal microscopy, which showed that liposomes (DiD‐labeled, blue) and macrophage membranes (DiR‐labeled, red) were successfully co‐localized (Figure S2A). Meanwhile, the successful fusion of lipo‐Mm membranes was confirmed by the change of zeta potential during the preparation process (Figure S2B). TEM and DLS showed that the prepared Dox@ML had a homogeneous spherical structure with a diameter of 113.7 ± 1.51 nm (Figure 2D and 2E). The hybrid membrane was encapsulated on the surface of siRNA@P by extrusion to form a nanoformulation called siRNA/Dox@PML.

Next, a CD24‐blocking peptide (GGGPLGLAG‐LDVFLYSE, M‐CSBP), responsive to MMP‐2, was DSPE‐PEG_2k_‐NHS to form a CD24 functional peptide (DSPE‐PEG_2k_‐M‐CSBP). The successful synthesis was confirmed by fourier‐transform infrared spectroscopy (FTIR) and nuclear magnetic resonance (NMR) (Figure 2F and S2C). DSPE‐PEG_2__k_‐M‐CSBP was anchored onto the surface of siRNA/Dox@PML through co‐incubation, yielding nanoformulation designated as siRNA/Dox@PMLC. DSPE‐PEG_2k_‐M‐CSBP binding resulted in a significant change in the zeta potential of siRNA/Dox@PMLC (Figure 2G). TEM results and hydrodynamic size show that the synthesized siRNA/Dox@PMLC has a uniform spherical structure with a size of about 133 nm (Figure 2H and 2I). DLS results showed that siRNA/Dox@PMLC exhibited excellent stability in PBS and culture medium supplemented with FBS (Figure 2J and Figure S2D).

In addition, sodium dodecyl sulfate‐polyacrylamide gel electrophoresis (SDS‐PAGE) was employed at each preparation stage to assess membrane proteins. The results showed that the protein bands of siRNA/Dox@PMLC were essentially the same as those of cell membrane (Figure 2K). No RNA degradation was detected during nanoformulation preparation as demonstrated by gel blocking experiments (Figure S2E). To assess the enzymatic responsiveness of M‐CSBP to the high level of MMP‐2 in the tumour environment (Zhang et al. 2023), we analysed the product of M‐CSBP reacting with MMP‐2 through liquid chromatography‐mass spectrometry (LC‐MS). As shown **in** Figure S2F and S2G, M‐CSBP was effectively cleaved after 3‐hourincubation with MMP‐2, yielding a new fragment LAG‐CSBP whose [M+H]^+^ ion was detected at m/z 1226.46 by mass spectrometry.

### 2.3 *In Vitro* Anti‐tumour Activity Studies of Nanoformulation and the Generation of Functionalized ApoBDs From Tumor Cells

**FIGURE 3:**
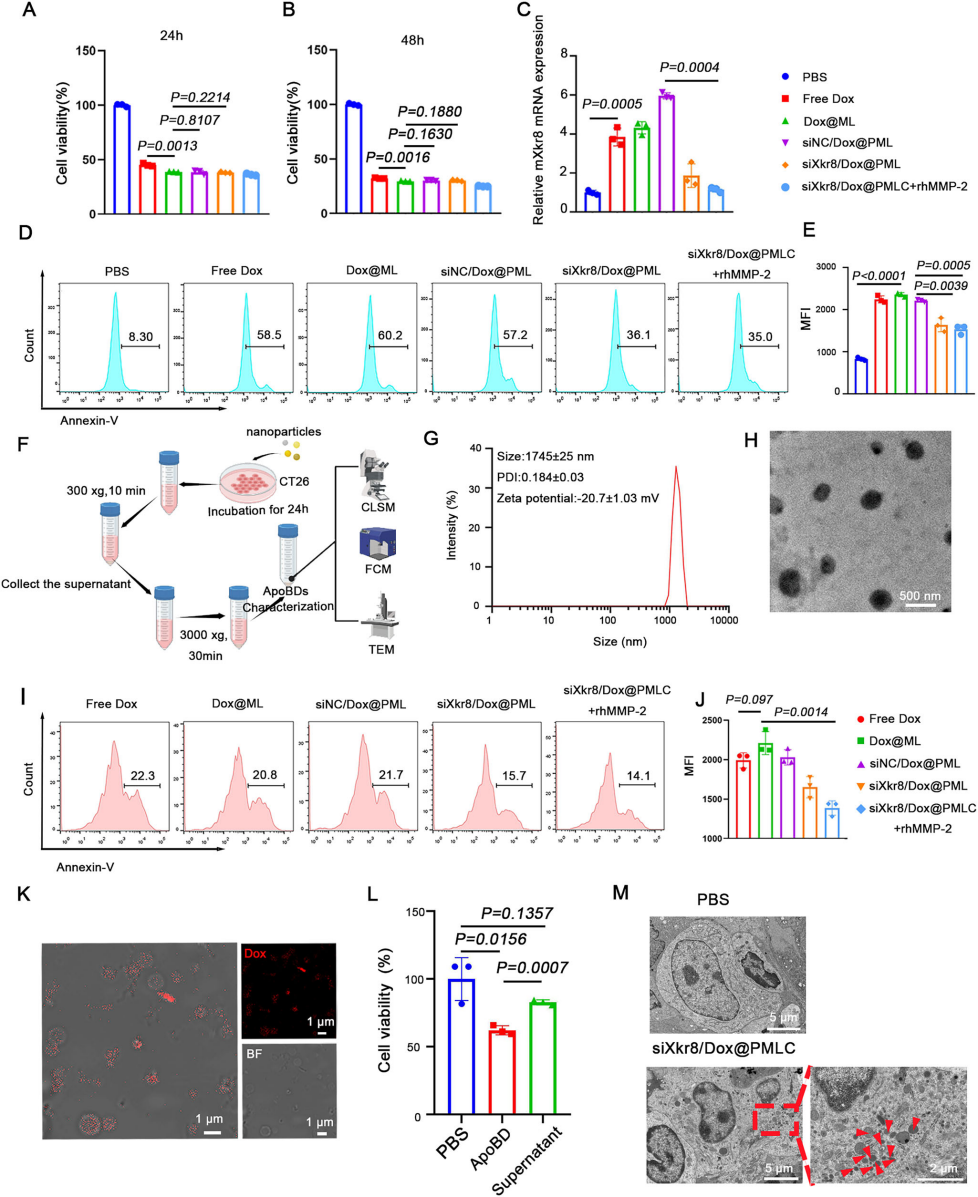
***In vitro* antitumor activity of nanoformulations and biological consequences of Xkr8 knockdown**. (A, B) Effects of various treatments on CT26 cell viability. CT26 cells were treated with PBS, Free Dox, Dox@ML, siNC/Dox@PML, siXkr8/Dox@PML, and siXkr8/Dox@PMLC+rhMMP‐2. Cell viability was assessed after treatment. (C) The relative Xkr8 expression levels in CT26 cells treated with different nanformulatioins were determined by qRT‐PCR. (D, E) Expression of PS on the cell surface was detected by flow assay after treatment of CT26 tumour cells with PBS, Free Dox, Dox@ML, siNC/Dox@PML, siXkr8/Dox@PML and siXkr8/Dox@PMLC+rhMMP2 for 24 h. (F) Schematic illustration of the isolation procedure for ApoBD. (G) Hydrated particle size distribution maps and zeta potential measurements of ApoBD. (H) TEM images of apoptotic vesicles. Scale bar: 500 nm. (I, J) PS expression on ApoBDs derived from treated CT26 cells. ApoBDs were isolated via differential centrifugation. PS expression on ApoBDs was then measured by flow cytometry. (*n* = 3, *P < 0.05, **P < 0.01, ***P < 0.001) (K) Confocal microscopy visualization of CT26 tumour cells releasing drug‐encapsulated ApoBD. Dox is shown in red. Scale bar: 1 µm. (L) CT26 cells were treated with siXkr8/Dox@PMLC. After centrifugation and separation, Dox@ApoBD, the corresponding supernatant was co‐cultured with CT26 cells. Tumor cell killing was observed after 48 h. (*n* = 3, *P < 0.05, **P < 0.01, ***P < 0.001) (M) TEM image of ApoBDs produced in *in vivo* tumour tissue.

To study the *in vitro* anti‐tumour activity of siXkr8/Dox@PMLC, we evaluated the viability of tumour cells treated with different nanoformulations by CCK‐8 method. As shown in Figures 3A and 3B, compared to the free Dox, the Dox@ML treated‐group showed significantly reduced cell viability, which could be attributed to the enhanced phagocytosis of the nanoformulation by CT26 cells due to the encapsulation with the biomimetic hybrid membrane. Moreover, similar cytotoxicity was observed in CT26 cells treated with siNC/Dox@PML and siXkr8/Dox@PML, indicating that siXkr8 itself exerted no cytotoxic effects. Since *Xkr8* is a key gene controlling PS exposure, we first verified the knockdown effect of siXkr8/Dox@PMLC on Xkr8 gene in CT26 by qRT‐PCR. As shown in Figure 3C, Dox could induce the up‐regulation of *Xkr8* in CT26 cells, which was consistent with previous literature (Chen et al. 2023), and the expression of mXkr8 gene was significantly down‐regulated in the cells when siXkr8 were introduced. Next, we quantified PS exposure on the cell surface by flow cytometry. When tumour cells were treated with siXkr8/Dox@PML, the exposure of PS on the cell surface was significantly inhibited compared to the nanoformulation containing control siRNA (siNC/Dox@PML). To mimic the TME, we pre‐treated siXkr8/Dox@PMLC with rhMMP‐2 and then incubated with tumour cells. The results showed that it had a similar effect to siXkr8/Dox@PML in inhibiting the exposure of PS on the surface of tumour cells (Figure 3D and 3E).

To verify whether siXkr8/Dox@PML could successfully induce the in situ generation of ApoBDs, we treated CT26 tumour cells with various formulations for 24 h. The corresponding ApoBDs were subsequently isolated using differential centrifugation and characterized by multiple methods (Figure 3F). DLS and Zeta potential analysis indicated that ApoBDs were spherical, approximately 1000 nm in size, with a zeta potential of ‐20.7 mV (Figure 3G and 3H). Next, we evaluated the exposure of PS on the surface of the ApoBDs. We treated tumour cells with free Dox and four nanoformulations (Dox@ML, siNC/Dox@PML, siXkr8/Dox@PML, and siXkr8/Dox@PMLC+rhMMP‐2). After treatment, ApoBDs were isolated by differential centrifugation and analysed for surface PS externalization by flow cytometry. As shown in Figures 3I and J, tumour cells treated with siXkr8/Dox@PML exhibited a significantly reduced level of PS exposure on the surface of ApoBDs. This result indicates that Xkr8 gene silencing can regulate PS flipping on the surface of ApoBDs. To further validate whether the primary ApoBDs efficiently encapsulated the Dox, we induced ApoBDs generation in tumour cells using the siXkr8/Dox@PMLC, followed by isolation via differential centrifugation. CLSM analysis confirmed the successful encapsulation of Dox within ApoBDs (Figure 3K). Meanwhile, tumour cells were treated with either ApoBDs or their corresponding supernatants. The CCK‐8 cytotoxicity assays revealed that the ApoBDs‐treated group exhibited significantly enhanced cell‐killing effects compared to the supernatant group (Figure 3L). The above experimental results can be attributed to the fact that after Dox in the nanoformulation induces apoptosis in tumour cells, the incompletely metabolized Dox can be encapsulated and stored within the ApoBDs secreted by tumour cells, rather than being released in free form into the supernatant. Furthermore, primary and secondary ApoBDs were sequentially isolated from tumour cells treated with siXkr8/Dox@PMLC. The ApoBDs were then co‐incubated with untreated CSCs, and cell viability was assessed by CCK‐8 assay. The results showed that secondary ApoBDs significantly reduced CSC viability, confirming their potent cytotoxic effect (Figure S3). Collectively, these findings demonstrate that ApoBDs not only serve as carriers for drug encapsulation and storage but also enable cascade transmission to effectively eliminate CSCs.

To evaluate the *in vivo* induction effect of the nanoformulation (siXkr8/Dox@PMLC) on the generation of ApoBDs, we established subcutaneous CT26 models in BALB/c mice. After 72 h of peritumoral injection of the siXkr8/Dox@PMLC, the tumour tissues were harvested for ultrastructural analysis. TEM observations revealed that typical ApoBDs structures were present in the tumour tissues of the siXkr8/Dox@PMLC treatment group (Figure 3M).

In conclusion, the above results indicate that the biomimetic nanoplatform can induce the in situ generation of Dox‐loaded primary ApoBDs in tumors and significantly inhibit the expression of PS on their surfaces.

### 2.4 The in Situ Generated ApoBDs Facilitated Immune Activation and Deep Drug Penetration

**FIGURE 4:**
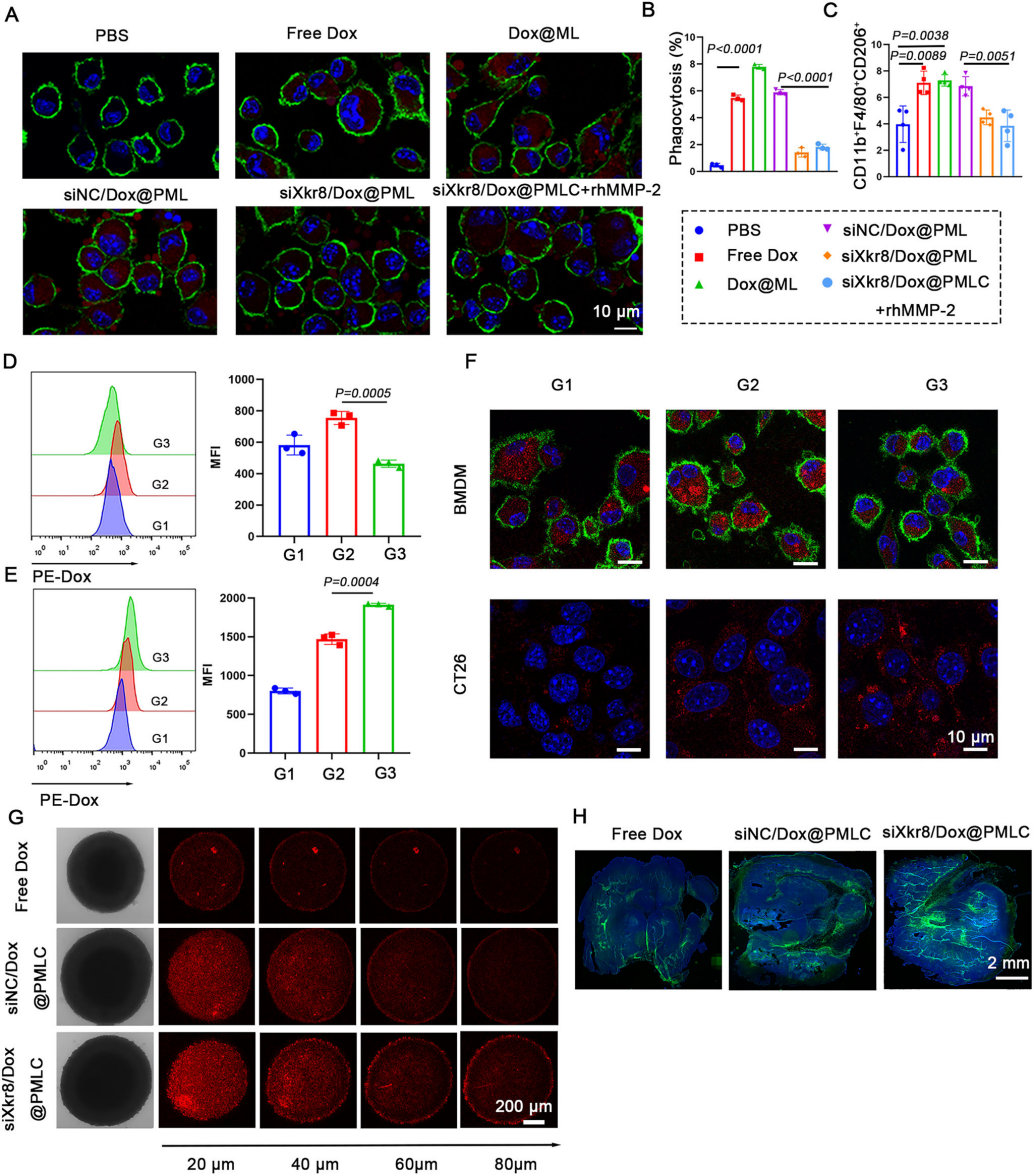
**Functionalized ApoBD‐mediated deep delivery**. (A) Confocal microscopy to observe phagocytosis of ApoBD by BMDM. BMDM were labeled with anti‐mCD11b (green), nuclei with hoechst (blue), and ApoBD (red). (B) Analysis of Phagocytosis of ApoBD by BMDM via flow cytometry. (C) Quantification of M2 phenotype BMDM. (D) Quantification of phagocytosis efficiency of BMDM on different ApoBDs. (E) Quantification of the phagocytosis efficiency of different ApoBDs by CT26 cells using flow cytometry. (F) Confocal microscopy observation of the uptake of ApoBD by CT26 and BMDM. Scale bar: 10 µm. (G) *In vitro* infiltration of tumour spheroids. *Z*‐axis scanning fluorescence images of CT26 tumour spheroids after incubation with Dox, siNC/Dox@PMLC, and siXkr8/Dox@PMLC for 24 h, respectively. Scale bar: 200 µm. (H) *In vivo* assessment of tumour tissue infiltration. Scale bar: 2 mm.

Studies have demonstrated that ApoBDs are more likely to be phagocytosed by macrophages via efferocytosis. During this process, PS exposed on the surface of ApoBDs serves as a critical “eat‐me” signal, playing a key role in recognition and uptake. Following efferocytosis, macrophages secrete immunosuppressive factors, promoting their polarization toward an M2‐like immunosuppressive phenotype. This mechanism ultimately exacerbates tumour immune evasion (Zhang et al. 2019). Therefore, we verified whether the nanoformulation‐induced generation of primary ApoBDs by tumour cells would result in less efficient phagocytosis by macrophages using a Transwell assay (Figure S4A). CLSM analysis revealed that compared to free Dox, Dox@ML, and siNC/Dox@PML, the siXkr8/Dox@PML and siXkr8/Dox@PMLC+rhMMP‐2 treatment groups significantly reduced the uptake of ApoBDs by BMDMs, indicating that these two nanoformulations markedly inhibited macrophage‐mediated efferocytosis (Figure 4A and S4C). Flow cytometry further confirmed that treatment with siXkr8/Dox@PML and siXkr8/Dox@PMLC+rhMMP‐2 resulted in an approximately 2.8‐fold decrease in the phagocytosis of ApoBDs by macrophages (Figure 4B and S4D). Based on the above findings, we quantified the proportion of M2‐like macrophages using flow cytometry. Notably, combination therapy with siXkr8 significantly suppressed the M2‐like polarization of macrophages (Figure 4C and S4E), thereby enhancing the anti‐tumour effect of macrophages. The above experimental results demonstrate that the primary ApoBDs induced in tumour cells by siXkr8/Dox@PMLC+rhMMP‐2 can evade phagocytic clearance by macrophages, significantly suppressing the polarization of macrophages toward the M2‐like immunosuppressive phenotype, consequently activating the anti‐tumour immune response.

Notably, ApoBDs significantly contribute to deep drug penetration in tumors. It has been reported in the literature that ApoBDs are capable of carrying residual drugs and delivering them to neighboring tumour cells after apoptosis (Zhao et al. 2021). However, the high level of PS exposure on the surface of ApoBDs leads to their rapid recognition and phagocytosis by macrophages. Based on this phenomenon, we hypothesized that suppressing PS exposure on ApoBDs could reduce macrophage clearance, enhance their accumulation at the tumour site, improve uptake by tumour cells, and thereby achieve deeper drug delivery. Thus, we developed a co‐culture system to investigate the transfer of different ApoBDs between tumour cells and BMDMs (Figure S4B). The uptake of these ApoBDs by tumour cells and BMDMs was quantified by flow cytometry. The results demonstrated that ApoBD with significantly reduced PS exposure (generated from siXkr8/Dox@PMLC‐treated tumour cells, G3 group) showed markedly lower phagocytic efficiency by BMDMs compared to those with higher PS exposure (produced by siNC/Dox@PMLC‐treated tumour cells, G2 group) (Figure 4D). Intriguingly, tumour cells themselves exhibited higher uptake efficiency toward ApoBDs (generated from siXkr8/Dox@PMLC‐treated tumour cells, G3 group) with reduced PS exposure (Figure 4E). Consistent with these findings, CLSM analysis revealed the same trend (Figure 4F). This phenomenon may be attributed to the weakened ability of BMDMs to recognize and phagocytose ApoBDs, and consequently increased opportunities for ApoBDs to interact with tumour cells, influencing intercellular communication within the TME. To further demonstrate the superiority of ApoBDs in delivering drugs in the TME, we constructed a heterogeneous tumour spheroid model. It was found that only a small amount of red fluorescence could be observed at the edge of the spheroid in the free Dox and siNC/Dox@PMLC groups, whereas the siXkr8/Dox@PMLC group demonstrated stronger penetration, with bright and widely distributed fluorescence observed even at a scanning depth of 80 µm (Figure 4G). To further validate the delivery efficiency in solid tumors, we conducted fluorescence imaging analysis of tumour tissues after peritumoral injection of the nanoformulations. Based on tumour tissue fluorescence imaging results, the green fluorescence of the siXkr8/Dox@PMLC was widely distributed throughout the tumour sections, exhibiting significantly higher intensity than that of the free Dox and siNC/Dox@PMLC (Figure 4H). Collectively, these findings demonstrate that suppression of PS exposure on ApoBDs significantly enhances their tumour‐penetrating capacity, enabling deep tissue delivery of therapeutic payloads within the TME.

These findings collectively indicate that ApoBDs generated in situ (with reduced exposure of PS) can evade phagocytic clearance by macrophages and disrupt immunosuppressive polarization. Additionally, ApoBDs generated in situ can enhance drug penetration into tumour tissues, offering an innovative therapeutic strategy for targeting and eradicating deep‐seated CSCs and enhancing antitumor efficacy.

### 2.5 siXkr8/Dox@PMLC Overcomes Immune Evasion in CSCs

**FIGURE 5:**
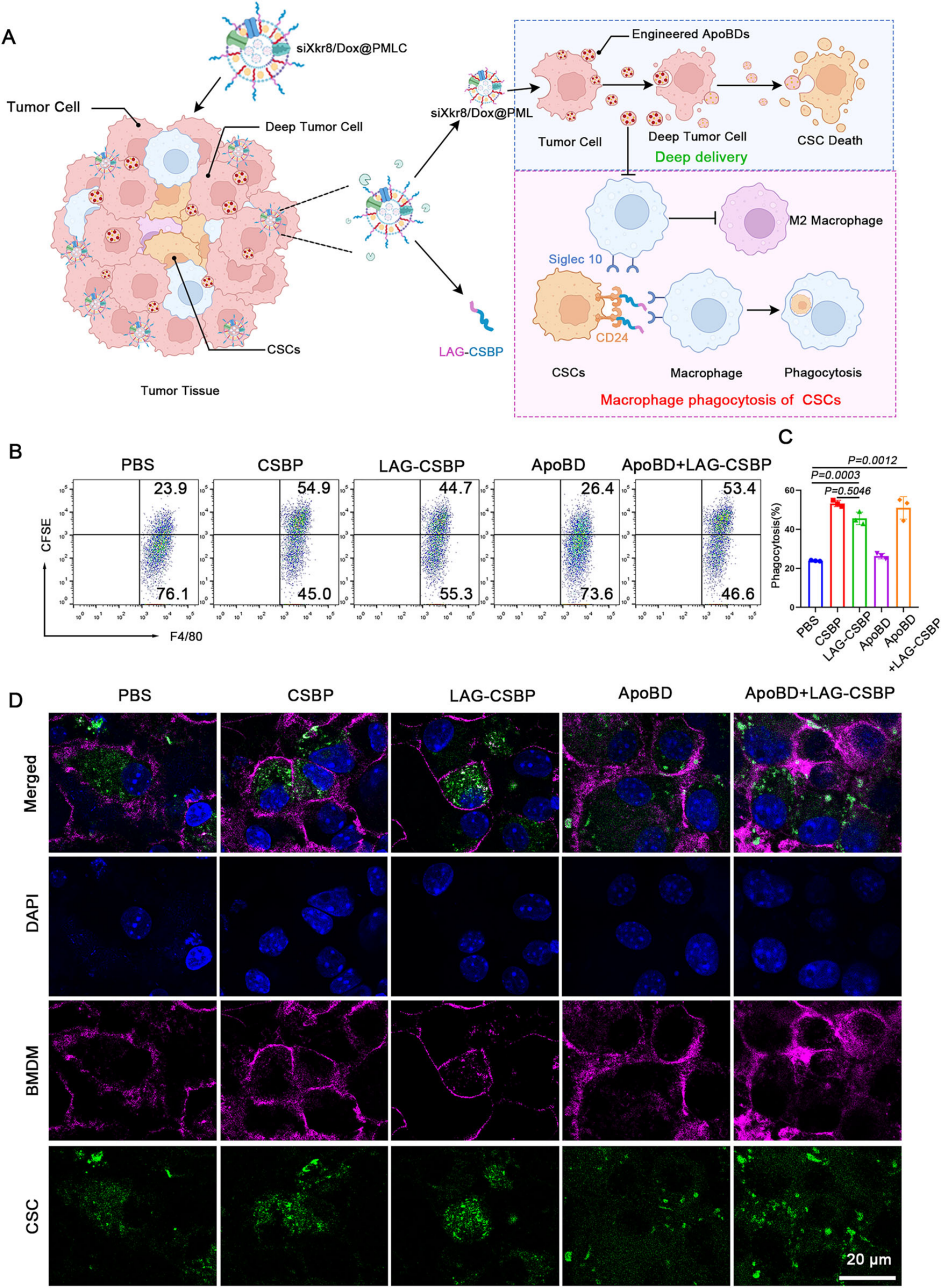
***In vitro* nanoformulations promote phagocytosis of macrophage cancer stem cells**. (A) Schematic representation of nanoformulation‐mediated phagocytic clearance of tumour stem cells by macrophages. (B, C) Phagocytic capacity of BMDMs toward CSCs. Representative flow cytometry dot plots (B) and corresponding quantitative statistical analysis (C) of the phagocytic efficiency across different treatment groups, *n* = 3. (D) Confocal microscopy images to observe the phagocytic effect of BMDMs on CSCs. BMDMs were labeled with anti‐mCD11b (purple), and CSCs were labeled with CFSE (green).

CSCs, owing to their distinctive self‐renewal capacity and multidrug resistance, can drive tumour recurrence and therapeutic failure even when only minimal residual populations remain after treatment. Previous studies have demonstrated that CSCs not only reside in deep tumour regions but also establish an effective immune evasion mechanism through the interaction between their overexpressed CD24 and macrophage Siglec‐10. In the aforementioned experiments, we have confirmed that in situ‐generated ApoBDs can significantly enhance drug penetration efficiency in deep tumour tissues. However, to achieve the specific elimination of CSCs, it is essential to overcome this critical immune evasion barrier (Figure 5A). To address this challenge, we innovatively combined an ApoBD‐mediated deep drug delivery system with CD24/Siglec‐10 signalling blockade. We treated CSCs with PBS, CD24‐blocking peptide (CSBP), LAG‐CSBP peptide released via enzymatic response, ApoBD (generated from siXkr8/Dox@PMLC‐treated tumour cells), and LAG‐CSBP + ApoBD. We then detected the phagocytosis efficiency of BMDMs toward CSCs. The results showed that LAG‐CSBP markedly promoted the phagocytosis of CSCs by BMDMs, confirming the functional activity of the enzyme‐responsive peptide released from the nanoformulation. Additionally, the combination of LAG‐CSBP and ApoBDs resulted in a marginally higher phagocytic efficiency than LAG‐CSBP treatment alone (Figure 5B and 5C). This can be attributed to the combined effects of the direct cytotoxicity of ApoBDs against CSCs and the synergistic blockade of the CD24/Siglec‐10 immune checkpoint by LAG‐CSBP. To visualize these results, we also used CLSM to observe the phagocytosis of CSCs by BMDMs. The confocal microscopy observations were consistent with the flow cytometry results, further verifying the phagocytosis‐promoting effect of LAG‐CSBP (Figure 5D).

### 2.6 Biodistribution Analysis *in Vivo*

Effective accumulation of nanoformulations at the tumour site is a prerequisite for their therapeutic effect. Therefore, we prepared DiR‐loaded nanoformulations (siRNA/DiR@PLC and siRNA/DiR@PMLC) and studied their biodistribution *in vivo*. Mice were imaged at different time points after intravenous administration. As shown in Figure S5A, siRNA/DiR@PMLC exhibited superior accumulation at the tumour site compared to the free DiR and siRNA/DiR@PLC. Significant tumour fluorescence was observed at 2 h post‐injection and persisted until 48 h. Then, we isolated the tumors and major organs and performed imaging analysis. In the siRNA/DiR@PMLC group, the fluorescence intensity in the tumour was significantly higher than that in the siRNA/DiR@PLC group (Figure S5B and S5C). This may be attributed to the increased *in vivo* circulation time of the nanoformulations due to the macrophage membrane, as well as their innate chemotactic ability toward the TMEs with inflammatory signatures (Cao et al. 2023; Zhou et al. 2023; Wu et al. 2022). These results suggest that the nanoformulations have superior tumour accumulation ability, which enhances drug delivery to the tumour site.

### 2.7 *In Vivo* Anti‐Tumor Activity Study of siXkr8/Dox@PMLC

**FIGURE 6:**
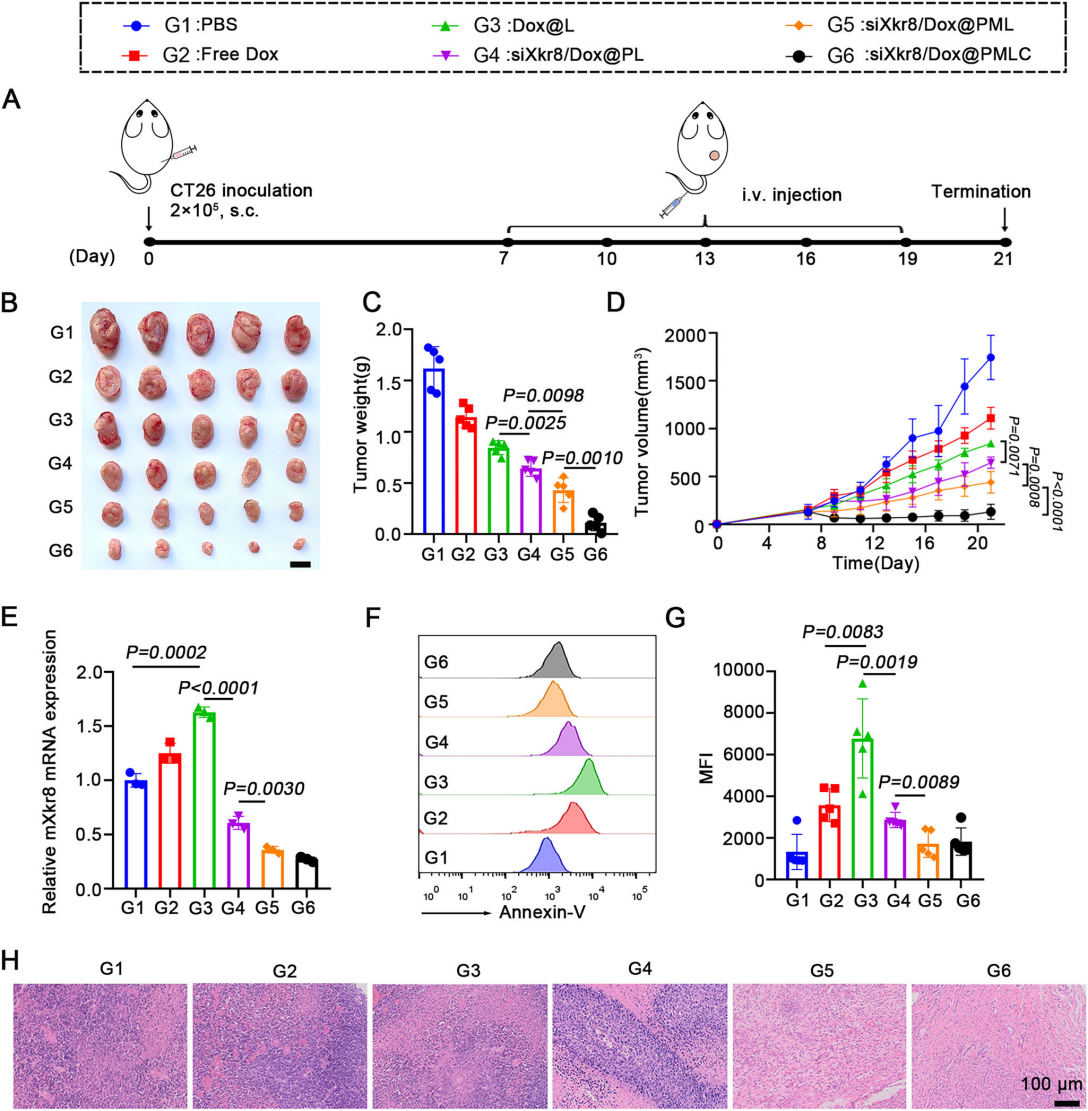
**Inhibition of CT26 tumour growth *in vivo* by siXkr8/Dox@PMLC**. (A) Schematic of the *in vivo* treatment protocol. (B) Representative images of subcutaneous CT26 tumors in mice. Scale bar: 1 cm. (C) Tumor weights of mice in different treatment groups. (D) Tumor growth curves during treatment, *n* = 5. (E) Expression of Xkr8 gene in tumour tissues. (F, G) The expression of PS on the cell surface was detected by flow cytometry. (H) H&E staining of tumors from loaded mice, scale bar: 100 µm.

In order to evaluate the anti‐tumour effect of siXkr8/Dox@PMLC *in vivo*, formulations (PBS, Free Dox, Dox@L, siXkr8/Dox@PL, siXkr8/Dox@PML and siXkr8/Dox@PMLC) were administered to mice bearing established subcutaneous CT26 colorectal tumors (Figure 6A). As shown in Figure 6B–D, the Dox@L treatment group exhibited a more significant tumour growth inhibitory effect compared to the free Dox group. More importantly, the siXkr8/Dox@PL group showed superior antitumor efficacy relative to Dox@L, which we attribute to siXkr8‐mediated reduction of PS exposure on tumour cells and tumour‐derived ApoBDs (Figure 6E–G). This facilitated both enhanced intratumoral drug penetration and reversal of PS‐mediated immunosuppression in the TME. Additionally, due to the active targeting of the macrophage membrane, the siXkr8/Dox@PML group demonstrated higher accumulation efficiency at the tumour site, further enhancing the antitumor effect. Notably, the siXkr8/Dox@PMLC group demonstrated the most potent tumour suppression among all treatment groups. Specifically, compared to the siXkr8/Dox@PML group, the anti‐tumour efficacy of the siXkr8/Dox@PMLC group was significantly enhanced, indicating that the CD24 blockade strategy can effectively enhance therapeutic efficacy. H&E staining of tumour tissue revealed that the siXkr8/Dox@PMLC‐treated group exhibited the most severe tumour tissue damage and the largest area of cell death, while no obvious damage was observed in the PBS group (Figure 6H). These data indicate that siXkr8/Dox@PMLC can effectively eliminate tumors.

### 2.8 *In Vivo* Immune Response

**FIGURE 7:**
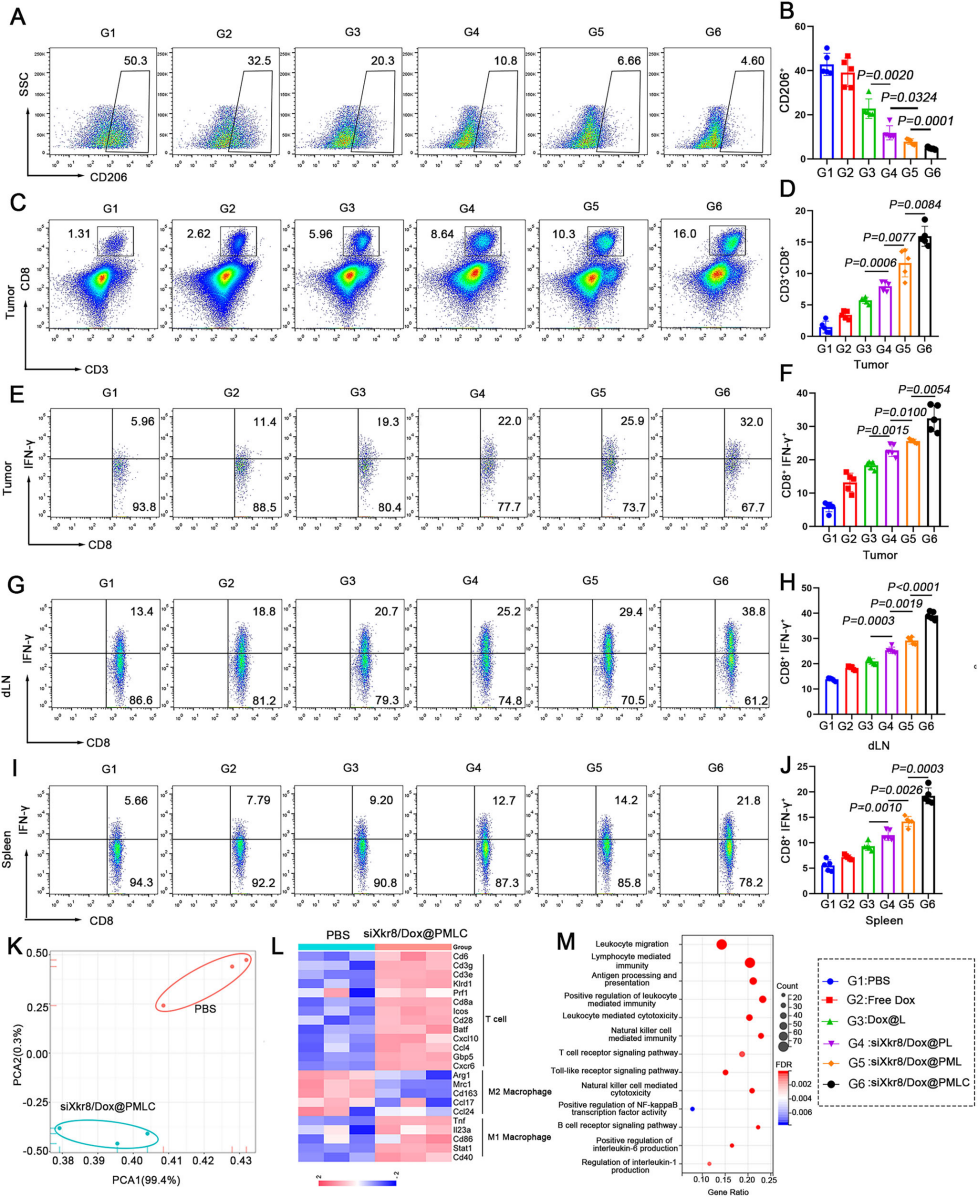
**siXkr8/Dox@PMLC activates anti‐tumour immune responses**. (A, B) The ratio of M1/M2 macrophages in tumors was assessed and quantified using flow cytometry. (C, D) Flow cytometry analysis of the proportion of CD8^+^ *T* cells in tumors from different treatment groups. (E, F) Flow representation of IFN‐γ^+^CD8^+^ T in tumors and quantitative statistics. (G, H) Flow cytometry analysis of representative maps and quantification of IFN‐γ^+^CD8^+^ *T* cells in dLN. (I, J) Representative maps and quantification of IFN‐γ^+^CD8^+^ *T* cells in spleen analysed by flow cytometry. (*n* = 5). (K) PCA plots of gene expression data obtained by RNA‐seq data, plotted for three biological replicates. (L) Heatmap of gene expression associated with immune cell types treated with PBS and siXkr8/Dox@PMLC. (M) Bubble plots of GO enrichment analysis for PBS and siXkr8/Dox@PMLC treatment.

To elucidate the anti‐tumour mechanisms of siXkr8/Dox@PMLC, we employed flow cytometry to analyse alterations in the immune cell composition within the TME and evaluate T cell activation in peripheral immune organs. The siXkr8/Dox@PMLC treatment group exhibited a significant reduction in the proportion of M2‐phenotype macrophages in tumour tissues compared to the PBS group (Figure 7A and 7B). Concurrently, the infiltration of CD8^+^ *T* cells was markedly increased in the siXkr8/Dox@PMLC group (Figure 7C and 7D), with a notable 6‐fold elevation in the proportion of CD8^+^ *T* cells expressing IFN‐γ (Figure 7E and 7F). Furthermore, siXkr8/Dox@PMLC significantly enhanced the capacity of CD8^+^ *T* cells in the draining lymph nodes and spleen to secrete IFN‐γ (Figure 7G‐7J). These findings together suggest that siXkr8/Dox@PMLC not only exerts local anti‐tumour effects but also stimulates systemic anti‐tumour immune responses.

To elucidate the mechanisms underlying tumour immunomodulation mediated by the nanoformulations in mice, we performed a transcriptomic analysis of tumour tissues from animals treated with siXkr8/Dox@PMLC. Principal component analysis (PCA) revealed that the variance of PCA1/PCA2 was less than 50%, demonstrating strong inter‐sample correlation and low intra‐group variability (Figure 7K). Sequencing analysis indicated that siXkr8/Dox@PMLC treatment significantly enhanced the enrichment of specific immune cell populations within the TME, particularly *T* cells and M1‐phenotype macrophages (Figure 7L). Gene ontology (GO) analysis identified significant enrichment of genes involved in immune‐related signalling pathways and cytokine signalling pathways (Figure 7 M). These findings demonstrate that siXkr8/Dox@PMLC therapy stimulates a powerful antitumor immune response.

### 2.9 siXkr8/Dox@PMLC Downregulates the Proportion of CSC to Suppress Lung Cancer Metastasis

**FIGURE 8:**
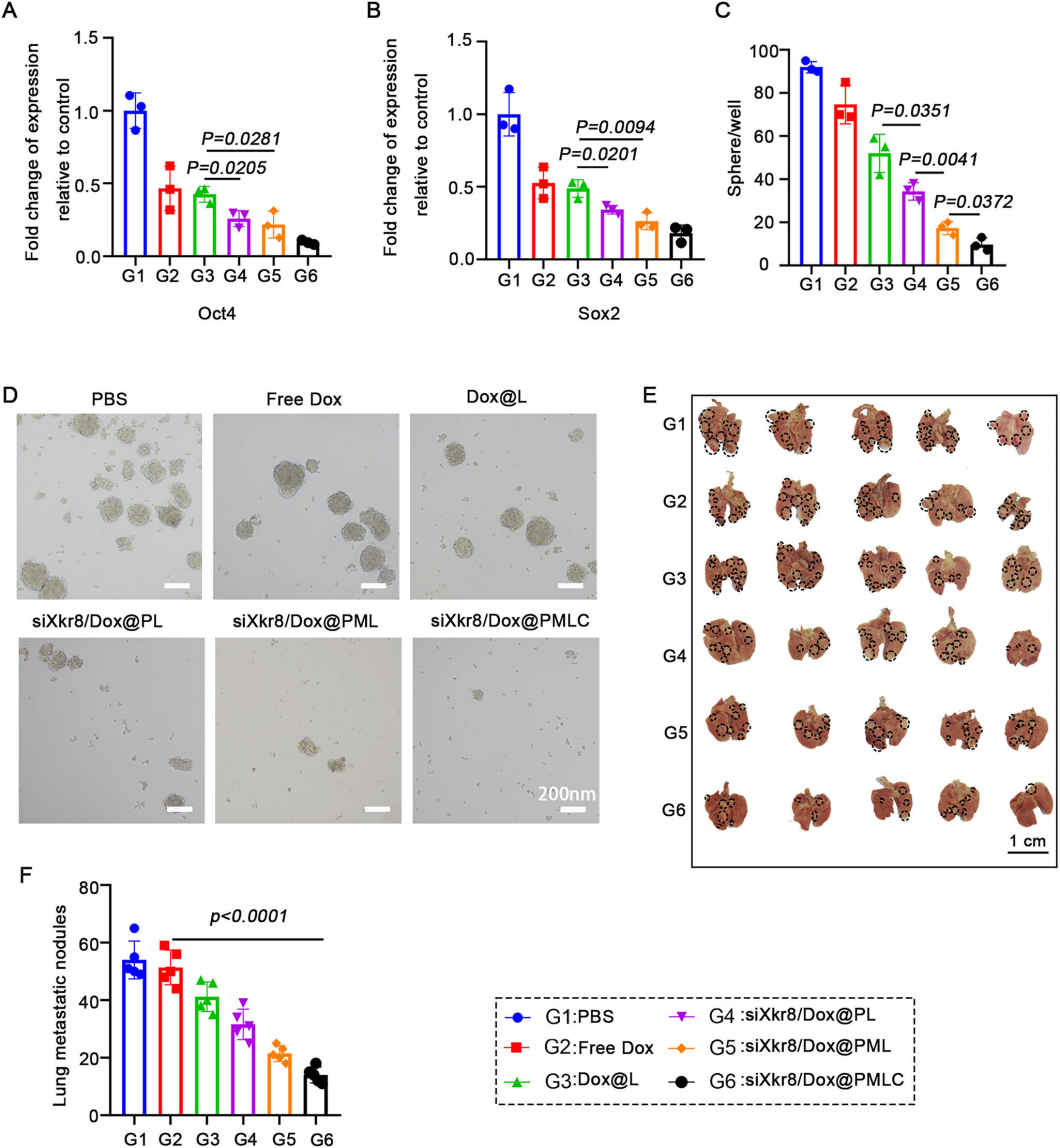
**siXkr8/Dox@PMLC treatment reduces the proportion of CSCs**. (A, B) qRT‐PCR analysis of gene expression related to cancer stem cells in tumour tissues, *n* = 3. (C, D) sphere formation assay. (E) Representative images of lung tissues front. Scale bar: 1 cm.(F) Statistical graph of lung nodules, *n* = 5.

To evaluate the impact of siXkr8/Dox@PMLC treatment on CSCs, we performed qRT‐PCR analysis of stemness‐associated genes (*Oct4, Sox2*) at the end of the treatment. Our findings demonstrated a significant downregulation of stemness‐related gene expression in the siXkr8/Dox @PMLC treatment group compared to PBS (Figure 8A and 8B). Meanwhile, the siXkr8/Dox@PMLC‐treated group exhibited the most substantial decrease in sphere forming ability (Figure 8C and 8D). This was consistent with the results of the aforementioned gene expression analysis, further confirming that the siXkr8/Dox@PMLC markedly suppressed CSC stemness. In the aforementioned experiments, we demonstrated that siXkr8/Dox@PMLC‐treated significantly reduced the proportion of CSCs in tumour tissues and suppressed their activity. To further investigate the impact of this treatment on the tumorigenic potential of CSCs, we conducted a limiting dilution tumorigenicity assay. CSCs pre‐treated with siXkr8/Dox@PMLC were mixed at varying proportions (0%, 5%, 10%, and 20%) with untreated CSCs and subcutaneously injected into mice, and their tumour‐forming capabilities were evaluated (Figure S6A). The results demonstrated that the tumour formation rate decreased significantly as the proportion of siXkr8/Dox@PMLC‐treated CSCs increased. In particular, tumour growth was significantly inhibited in mice implanted with 20% siXkr8/Dox@PMLC‐treated CSCs (Figure S6B and S6C). Meanwhile, qRT‐PCR detected the lowest expression of stemness‐related genes in the tumour tissues of mice with 20% drug‐treated CSCs (Figure S6D–F). These results were consistent with those of the *in vitro* sphere forming experiments, further verifying that siXkr8/Dox@PMLC had a significant inhibitory effect on the tumour forming ability of CSCs. These findings suggest that the siXkr8/Dox@PMLC not only reduces the abundance of CSCs but also markedly impairs their tumour‐initiating capacity, highlighting the potential of siXkr8/Dox@PMLC as a promising strategy to suppress CSCs activity.

Studies have shown that CSCs play a key role in mediating lung metastasis (Chu et al. 2024). Therefore, we further evaluated the anti‐metastatic potential of siXkr8/Dox@PMLC. Comparative analysis of the metastatic nodules in the lungs across different treatment groups revealed a significantly higher number of nodules in the PBS control group compared to all other groups. Conversely, the siXkr8/Dox@PMLC group showed a substantial reduction in lung metastatic nodules, significantly fewer than the free Dox treatment group (Figure 8E and 8F), strongly indicating the potent anti‐metastatic effect of siXkr8/Dox@PMLC. In addition, H&E staining of lung tissues further confirmed this result (Figure S7). Collectively, these results underscore the critical role of siXkr8/Dox@PMLC in suppressing CSC proliferation and inhibiting lung metastasis.

### 2.10 Biosafety Assessment

To evaluate the biosafety profile of the nanoformulation, we monitored the body weight of mice throughout the treatment period and observed no significant alterations in body weight (Figure S8A). Furthermore, H&E staining of major organs (heart, liver, spleen, lungs, and kidneys) collected at the end of the treatment revealed no apparent pathological damage (Figure S8B). To further assess the biosafety of the siXkr8/Dox@PMLC, we conducted comprehensive hematological and biochemical analyses. The results demonstrated no significant differences in white blood cell (WBC) count, haemoglobin (HGB) level, red blood cell (RBC) count, or platelet (PLT) count between the siXkr8/Dox@PMLC treatment group and the PBS control group (Figure S9A–D), indicating the absence of hematotoxicity associated with siXkr8/Dox@PMLC. Additionally, blood biochemical analysis revealed no significant differences in aspartate aminotransferase (AST), alanine aminotransferase (ALT), urea (UREA), or creatinine (CREA) levels between the siXkr8/Dox@PMLC treatment group and the PBS group (Figure S9E–H), suggesting that the nanoformulations did not induce any notable hepatotoxicity or nephrotoxicity. These findings collectively confirm the safety of the nanoformulations in normal organs, supporting their potential for therapeutic application.

## 3 Conclusions

In this study, we developed a biomimetic “core‐shell” nanoplatform (siXkr8/Dox@PMLC) that integrates cascade‐amplified drug delivery, immune escape signal intervention, and remodelling of the tumour immune microenvironment. The nanoplatform enables effective elimination of deeply located CSCs while synergistically enhancing the efficacy of tumour immunotherapy. The siXkr8/Dox@PMLC can induce tumour cells to in situ generate drug‐loaded primary ApoBDs, forming a cascade‐amplified drug delivery system. Compared to traditional exogenous preparation of EVs, these drug‐loaded vesicles generated in situ using tumour cells as “bioreactors” retain the membrane protein composition of the source cells. They exhibit excellent biocompatibility and homologous targeting capabilities, avoiding issues inherent in conventional exogenous methods such as significant batch‐to‐batch variability, membrane protein damage, and complex separation and purification processes (Zhang et al. 2025; Xu et al. 2025). Furthermore, in situ‐generated ApoBDs respond to the TME by continuously releasing drugs, enabling deep penetration and prolonged retention. This overcomes the limitation of poor drug permeability within solid tumors.

Meanwhile, the inhibition of PS exposure on the surface of ApoBDs mediated by siXkr8 can effectively reshape the immune phenotype of macrophages, laying a foundation for subsequent immune clearance. On this basis, the CD24‐blocking peptide released by siXkr8/Dox@PMLC in the TME can specifically block the CD24/Siglec‐10 immune checkpoint signalling pathway, further enhancing the ability of macrophages to recognize and phagocytose CSCs. More importantly, *in vivo* experiments have confirmed that siXkr8/Dox@PMLC can inhibit tumour growth and reduce the proportion of CSCs in tumour tissues. In general, these research findings indicate that the multi—mechanism synergy mediated by siXkr8/Dox@PMLC not only significantly inhibits tumour growth but also provides an innovative solution for eliminating CSCs, showing remarkable potential for clinical translation.

## 4 Materials

### 4.1 Cell and Spheres Culture

CT26, NIH‐3T3 were cultured in DMEM medium (Gibco). The CSCs were enriched using a serum‐free suspension culture method. CT26 cells were inoculated into 6‐well ultralow attachment plates with serum‐free DMEM/F12 (Gibco) culture media supplemented with epidermal growth factor, basic fibroblast growth facto, N2 additives and B27 additives. After three days of incubation under the specified conditions, each well was supplemented with 1 mL of the above medium. On the seventh day, passaging was performed, and the mammospheres formed on the fourteenth day was used for subsequent experiments.

### 4.2 Agarose Gel Electrophoresis Assay

First, 200 µg of PAMAM was dissolved in 94 µL of PBS buffer solution to obtain a master mix with a concentration of 2.12 µg/µL. At the same time, prepare a stock solution at a concentration of 0.265 µg/µL. Subsequently, the PAMAM solution was mixed with siRNA solution at different mass ratios of PAMAM/siRNA (0:1, 5:1, 10:1, 20:1, 40:1, 80:1), and incubated for 30 min at 300 rpm/min at room temperature to ensure the full binding of siRNA and PAMAM. After the incubation was completed, 10×loading Buffer was added to the samples and mixed thoroughly. Load the sample into each gel well for electrophoresis.

### 4.3 Extraction of Macrophage Membranes

First, RAW 264.7 cells were collected and centrifuged at 3500 rpm for 10 min. After discarding the supernatant, the cells were washed again with PBS, counted, and resuspended at 1×10^7^ cells per aliquot in CER reagent supplemented with 1% PMSF for lysis on ice. The lysate was subjected to 2–3 cycles of freeze‐thaw in liquid nitrogen, followed by homogenization with a pre‐cooled glass homogenizer. After low‐speed centrifugation, the supernatant was ultracentrifuged at 13,000 rpm (4°C, 30 min) to pellet membranes.

### 4.4 Preparation of siRNA/Dox@PMLC

Cell membrane precipitates were resuspended in PBS and mixed with Dox@L liposomes at a 1:20 mass ratio based on protein content. Hybrid nanoformulation (Dox@ML) were prepared by extruding the mixture 11 times through a 400 nm polycarbonate membrane. Subsequently, siRNA@P was added at a 1:15 mass ratio and extruded sequentially through 400 and 200 nm membranes to form siRNA/Dox@PML nanoformulation. Finally, DSPE‐PEG2k‐M‐CSBP peptide was incorporated into the nanoformulation suspension via stirring, and purified by centrifugation at 13,000 rpm to remove excess peptide, yielding the final siRNA/Dox@PMLC nanoformulation.

### 4.5 MMP‐2 Enzyme Response Assay

First, 2 mg of siRNA/Dox@PMLC nanoformulation were weighed and dissolved in 3 mL of TCNB. The siRNA/Dox@PMLC solution was subsequently co‐incubated with activated MMP‐2 enzyme for 3 h (37°C). The supernatant was collected after centrifugation and analysed by mass spectrometry to evaluate MMP‐2‐responsive release of the nanoformulation. M‐CSBP peptide and DSPE‐PEG_2k_‐NHS were mixed at a 2:1 mass ratio in DMF. The peptide solution was slowly added to the DSPE‐PEG_2k_‐NHS under stirring, and triethylamine was used to adjust the pH to 8.0–9.0. The reaction proceeded for 4–6 h at room temperature in the dark. The mixture was then diluted with eight volumes of water and dialyzed (MWCO 3500 Da) for two days to remove unreacted compounds. The purified product was freeze‐dried and stored at –20°C for further use.

### 4.6 Analysis of Xkr8 mRNA Levels by qRT‐PCR

CT26 cells were treated with the indicated compounds for 24 h. Total RNA was extracted and reverse‐transcribed into cDNA. qRT‐PCR was performed using SYBR Green, with GAPDH as the endogenous control. Gene expression was quantified using the ΔΔCT method. siRNA and primer sequences are provided in Supplementary Tables S1 and S2, respectively.

### 4.7 Detection of PS Exposure on the Cell Surface

CT26 cells were seeded in plates and treated with the formulations (PBS, Free Dox, Dox@ML, siNC/Dox@PML, siXkr8/Dox@PML, or siXkr8/Dox@PMLC+rhMMP‐2) for 24 h. After washing with PBS, cells were resuspended in binding buffer, stained with Annexin‐V antibody on ice in the dark, and analysed by flow cytometry.

### 4.8 Preparation and Characterization of ApoBDs

CT26 cells were treated with nanoformulation for 24 h to induce apoptosis. Subsequently, the culture supernatant and cells were collected, transferred to a 15 mL centrifuge tube, and centrifuged at 500 g for 10 min at 4°C to remove cells and cell debris. Next, the supernatant was centrifuged at 3000 g for 30 min at 4°C to obtain ApoBDs precipitates. The morphology was assessed by Transmission electron microscopy (TEM) (HT7700, Hitachi, Japan), Diameter and Zeta potential of apoptotic apoptpyic were measured by dynamic light scattering (DLS) (Zetasizer Nano S90, Malvern, UK). Confocal laser scanning microscope (CLSM) detected the encapsulation of Dox in ApoBDs.

Detection of ApoBDs surface PS expression by flow cytometry. CT26 cells were treated with different groups of PBS, Free Dox, Dox@ML, siNC/Dox@PML, siXkr8/Dox@PML, siXkr8/Dox@PMLC+rhMMP‐2 for 24 h. Subsequently, ApoBDs were isolated, and ApoBDs precipitates were resuspended by adding 1×Binding Buffer. At the same time, Annexin‐V was added to each tube and mixed well, and then flow cytometry was performed.

### 4.9 Transwell

CT26 cells were seeded in chambers and treated with PBS, Free Dox, Dox@ML, siNC/Dox@PML, siXkr8/Dox@PML and siXkr8/Dox@PMLC+rhMMP‐2 groups for 4 h. After removing uninternalized nanoformulation by PBS washing, the chambers were transferred to fresh DMEM‐coated plates and cultured for another 24 h. BMDM were then collected for analysis of apoptotic vesicle phagocytosis and M2 macrophage polarization by flow cytometry and confocal microscopy.

### 4.10 Culture of Multicellular Tumor Spheres

Multicellular heterogeneous tumour spheres were constructed based on previous reports. First, 80 µL of 2.0% agarose solution was added to a 96‐well plate and cooled to room temperature. The isolated BMDM was mixed with CT26 and NIH‐3T3 cells in a ratio of 4:1:4 and inoculated into the 96‐well plate. The total number of inoculated cells in each well was maintained at 5000 cells and cultured for 7 days to form multicellular tumour spheres. To induce macrophage differentiation, the medium was supplemented with 50 ng/mL macrophage colony‐stimulating factor.

### 4.11 ApoBDs Mediate Deep Delivery of Drugs

Free Dox, siNC/Dox@PMLC and siXkr8/Dox@PMLC nanoformulations were co‐cultured with multicellular heterogeneous tumorspheres induced up to day 7 in 96‐well plates for 24 h. Tumorspheres were removed from the wells, excess carriers were washed away with PBS, and fluorescence of Dox was evaluated by laser confocal microscopy using fluorescence of Dox as a characteristic indicator to the surface of the tumorspheres as 0 µm, and Z‐axis scanning and imaging were performed layer by layer every 20 µm. In mice bearing subcutaneous CT26 tumors, Free Dox, siNC/Dox@PMLC, and siXkr8/Dox@PMLC were injected peritumorally. After 48 h, tumors were harvested for sectioning and whole‐slide scanning to assess fluorescence distribution.

### 4.12 Co‐Culture Experiments

CT26 cells were seeded on one slide, while induced BMDM were adjusted to an appropriate density and seeded on another slide. The above two slides were placed side by side in a medium‐sized Petri dish. Subsequently, apoptotic vesicles produced by CT26 cells treated with different nanoformulations (Free Dox, siNC/Dox@PMLC, siXkr8/Dox@PMLC) were isolated and added into the co‐culture system of CT26 and BMDM, respectively, and co‐cultivated for 24 h. At the end of the culture, the slides inoculated with CT26 and BMDM were removed for observation and quantitative analysis by confocal microscopy and flow cytometry, respectively.

### 4.13 Phagocytosis Assay of CSCs

To the CFSE‐stained CSCs, 100 µL of different nanoformulations were added: the PBS group, CSBP (final concentration of 400 µM), LAG‐CSBP peptide released by enzymatic response, ApoBDs, and LAG‐CSBP + ApoBDs, and the nanoformulations were gently shaken to ensure that the nanoformulations were well mixed with the CT26 cells. The cells were placed on ice and incubated for 30 min. 500 µL of BMDM at a density of 1×10^5^ cells/mL was subsequently mixed with the above co‐incubation system, shaken and mixed well, and the co‐incubation system was then spread in a 24‐well plate and placed in an incubator for overnight incubation. At the end of the co‐culture, the cells were collected for flow cytometry and confocal detection.

### 4.14 Biodistribution

BALB/c mice were subcutaneously inoculated with CT26 cells on the right back. When tumour volume reached 80–100 mm^3^, mice received intravenous injections of siRNA/DiR@PMLC, siRNA/DiR@PLC, and Free DiR (5 mg/kg DiR). Nanoformulation distribution was monitored using an IVIS imaging system at multiple time points.

### 4.15 In Vivo Anti‐Tumor Studies

Subcutaneous tumors were established in BALB/c mice by inoculating 2×10^5^ CT26 cells. Upon reaching 80–100 mm^3^, mice were randomized into six groups receiving PBS, Free Dox, Dox@L, siXkr8/Dox@PL, siXkr8/Dox@PML, or siXkr8/Dox@PMLC via tail vein injection every three days (five total doses). Dox was administered at a dose of 0.5 mg/kg, siXkr8 was administered at a dose of 1 mg/kg, and DSPE‐PEG_2k_‐M‐CSBP was administered at a dose of 0.2 mg/kg. Tumor dimensions were periodically measured, and volume was calculated as V = L(Length)×W^2^(width) ×0.5 (*n* = 5 per group).

### 4.16 Establishment of the Lung Metastasis Model

Single‐cell suspensions were prepared from treated tumors by enzymatic digestion, adjusted to 5×10^5^ cells/mL, and 200 µL was intravenously injected into BALB/c mice. Twenty days after the injection, the mice were euthanized and the bilateral lung tissues were removed intact. The lung tissues were placed in medium fixation, and the number of metastatic nodules in the lungs was observed and counted. Subsequently, the fixed lung tissue samples were sent to Servicebio for paraffin embedding, sectioning and H&E staining to analyse the lung metastases.

### 4.17 Statistical Analysis

Data were analysed with Microsoft Excel, Avantage and GraphPad Prism 9. Data were represented as mean ± standard deviation (SD) and *n* = 3 unless otherwise indicated. Differences between two groups were examined by single‐tailed Student's t‐test. The significance was indicated by *P* < 0.05 (*P < 0.05, **P < 0.01 and ***P < 0.001), and *P* > 0.05 indicated no significant difference (ns).

## Conflicts of Interest

The authors declare no conflicts of interest.

## Supporting information




**Supporting Figure S1**: (A) TEM image of siRNA@PAMAM and (B) the results of hydrated particle size. Scale bar: 50 nm. **Figure S2**: (A) Confocal microscopy images of Lipo and Mm co‐localization. (Lipo labeled with DiD, blue; Mm labeled with DiR, red; scale bar: 2 µm) (B) Zeta potentials of Dox@L and Dox@ML, *n* = 3. (C) ^1^H nuclear magnetic resonance (NMR) spectra of M‐CSBP, DSPE‐PEG‐NHS and DSPE‐PEG‐M‐CSBP. (D) Stability of siRNA/Dox@PMLC in culture medium supplemented with FBS, *n* = 3. (E) Agarose gel electrophoresis of siXkr8@P, siXkr8/Dox@PML, siXkr8/Dox@PMLC. (F‐G) The liquid phase absorption peaks and the corresponding mass spectra of siXkr8/Dox@PMLC after treatment with MMP‐2. **Figure S3**: Apoptotic bodies exhibit toxicity toward CSCs. (*n* = 3, *P < 0.05, **P < 0.01, ***P < 0.001). **Figure S4**: (A) Schematic diagram of Transwell setup. CT26 cells were initially co‐cultured with various nanoparticles in the upper chamber for 4 h. Thereafter, nanoparticles that were not phagocytosed by CT26 cells were removed from the upper chamber, and the incubation was continued for an additional 20 h. During this period, the produced Dox@ApoBD permeated through the microporous filter membrane into the lower chamber, which was pre‐seeded with BMDM. (B) Schematic diagram of the co‐culture of functionalized ApoBD with CT26 cells and BMDM. (C) Representative flow cytometry plot of Dox@ApoBD phagocytosis by BMDM. (D) Representative flow cytometry plot of M2 phenotype macrophages. **Figure S5**: ***In vivo* distribution of nanoparticles**. (A) Fluorescence imaging of mice at 1, 2, 4, 8, 12, 24, 36 and 48 h after injection. (B) Fluorescence images of mouse tumors and major organs. (C) Quantitative analysis of fluorescence intensity of isolated images. (*n* = 3, *P < 0.05, **P < 0.01, ***P < 0.001). **Figure S6**: **siXkr8/Dox@PMLC treatment significantly inhibits the tumorigenicity of CSCs *in vivo***. (A) Schematic illustrstion of tumorigenicity of tumour stem cells. (B) *In vitro* tumour images of different treatment groups, *n* = 5. (C) Tumor weights of mice in different treatment groups, *n* = 5. (D‐F) Expression of relevant stemness genes in tumour tissues, *n* = 3. **Figure S7**: Representative H&E staining of lung tissue metastases. **Figure S8**: **Nanoparticle biocompatibility assessment**. (A) Change in body weight of mice during treatment. (B) H&E staining of heart, liver, spleen, lungs and kidneys in the mice of different groups. Scale bar: 100 µm. **Figure S9**: Blood physiology and biochemistry index of mice injected with indicated drugs, *n* = 5. **Table S1**: siRNA sequences. **Table S2**: Primers sequences for real time qRT‐PCR.

**Supporting Tables**: jev270292‐sup‐0002‐tablesS1‐S2.docx

**Supporting Figure**: jev270292‐sup‐0003‐figureS1.tif

**Supporting Figure**: jev270292‐sup‐0004‐figureS2.tif

**Supporting Figure**: jev270292‐sup‐0005‐figureS3.tif

**Supporting Figure**: jev270292‐sup‐0006‐figureS4.tif

**Supporting Figure**: jev270292‐sup‐0007‐figureS5.tif

**Supporting Figure**: jev270292‐sup‐0008‐figureS6.tif

**Supporting Figure**: jev270292‐sup‐0009‐figureS7.tif

**Supporting Figure**: jev270292‐sup‐0010‐figureS8.tif

**Supporting Figure**: jev270292‐sup‐0011‐figureS9.tif

## Data Availability

Research data are not shared.
