# Supplementary material for: In Situ Engineered “Cascade‐Amplified” Drug‐Loaded Vesicles for Enhanced Cancer Stem Cell Therapy
Source: J Extracell Vesicles. 2026 May 9;15(5):e70292. doi: 10.1002/jev2.70292 (PMC13157588; doi:10.1002/jev2.70292)
Supplement: Supplementary file 1 — Supporting Figure S1: (A) TEM image of siRNA@PAMAM and (B) the results of hydrated particle size. Scale bar: 50 nm. Figure S2: (A) Confocal microscopy images of Lipo and Mm co‐localization. (Lipo labeled with DiD, blue; Mm labeled with DiR, red; scale bar: 2 µm) (B) Zeta potentials of Dox@L and Dox@ML, n = 3. (C) 1H nuclear magnetic resonance (NMR) spectra of M‐CSBP, DSPE‐PEG‐NHS and DSPE‐PEG‐M‐CSBP. (D) Stability of siRNA/Dox@PMLC in culture medium supplemented with FBS, n = 3. (E) Agarose gel electrophoresis of siXkr8@P, siXkr8/Dox@PML, siXkr8/Dox@PMLC. (F‐G) The liquid phase absorption peaks and the corresponding mass spectra of siXkr8/Dox@PMLC after treatment with MMP‐2. Figure S3: Apoptotic bodies exhibit toxicity toward CSCs. (n = 3, *P < 0.05, **P < 0.01, ***P < 0.001). Figure S4: (A) Schematic diagram of Transwell setup. CT26 cells were initially co‐cultured with various nanoparticles in the upper chamber for 4 h. Thereafter, nanoparticles that were not phagocytosed by CT26 cells were removed from the upper chamber, and the incubation was continued for an additional 20 h. During this period, the produced Dox@ApoBD permeated through the microporous filter membrane into the lower chamber, which was pre‐seeded with BMDM. (B) Schematic diagram of the co‐culture of functionalized ApoBD with CT26 cells and BMDM. (C) Representative flow cytometry plot of Dox@ApoBD phagocytosis by BMDM. (D) Representative flow cytometry plot of M2 phenotype macrophages. Figure S5: In vivo distribution of nanoparticles. (A) Fluorescence imaging of mice at 1, 2, 4, 8, 12, 24, 36 and 48 h after injection. (B) Fluorescence images of mouse tumors and major organs. (C) Quantitative analysis of fluorescence intensity of isolated images. (n = 3, *P < 0.05, **P < 0.01, ***P < 0.001). Figure S6: siXkr8/Dox@PMLC treatment significantly inhibits the tumorigenicity of CSCs in vivo . (A) Schematic illustrstion of tumorigenicity of tumour stem cells. (B) In vitro tumour images of different treatme [file JEV2-15-e70292-s004.docx]

Title：In Situ Engineered “Cascade-Amplified” Drug-Loaded Vesicles for Enhanced Cancer Stem Cell Therapy

*Tiantian Zhang^1,3^, YuanYuan Wei^1^, Zimai Liu^1^, Zixian Wu^1^, Xiaoxi Wang^1^, Kai Li^1^, Hui Liu^1^, Jiao Lu^1^, Qianxi Lu^1^, Meiyi Liu^1^, Yongchao Wang^1,4*^, Zhenzhen Chen^1,2*^*

^1^School of Life Sciences, Zhengzhou University, Zhengzhou 450001, China.

^2^School of Medicine, Shihezi University, Shihezi 832000, China

^3^School of Materials Science and Engineering, Zhengzhou University, Zhengzhou 450001, China

^4^Longhu Laboratory of Advanced Immunology, Zhengzhou 450046, China

Corresponding Authors:

* Yongchao Wang, E-mail: wangyongchao@zzu.edu.cn

* Zhenzhen Chen, E-mail: chenzz2015@zzu.edu.cn


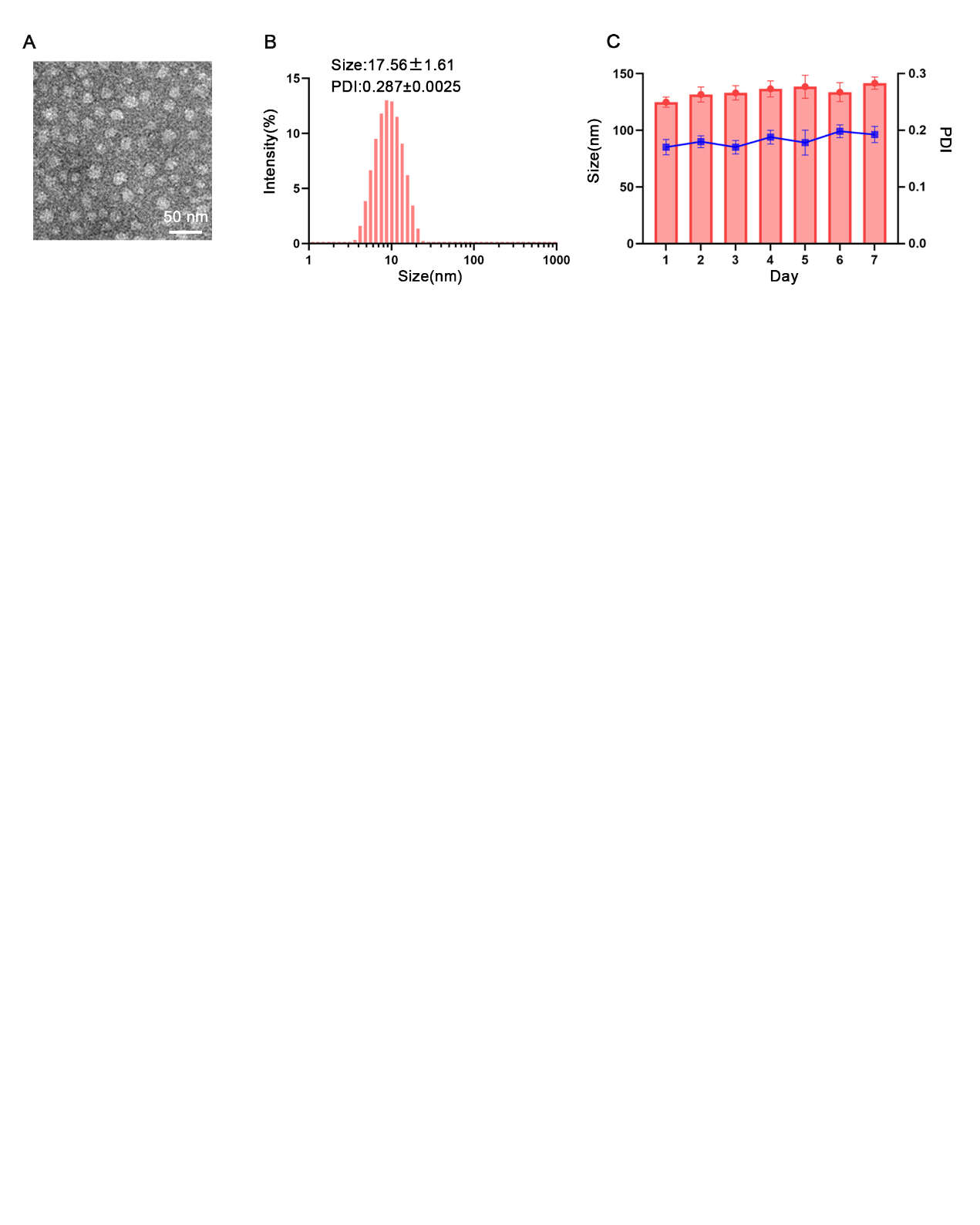


**Figure S1** (A) TEM image of siRNA@PAMAM and (B) the results of hydrated particle size. Scale bar: 50 nm.


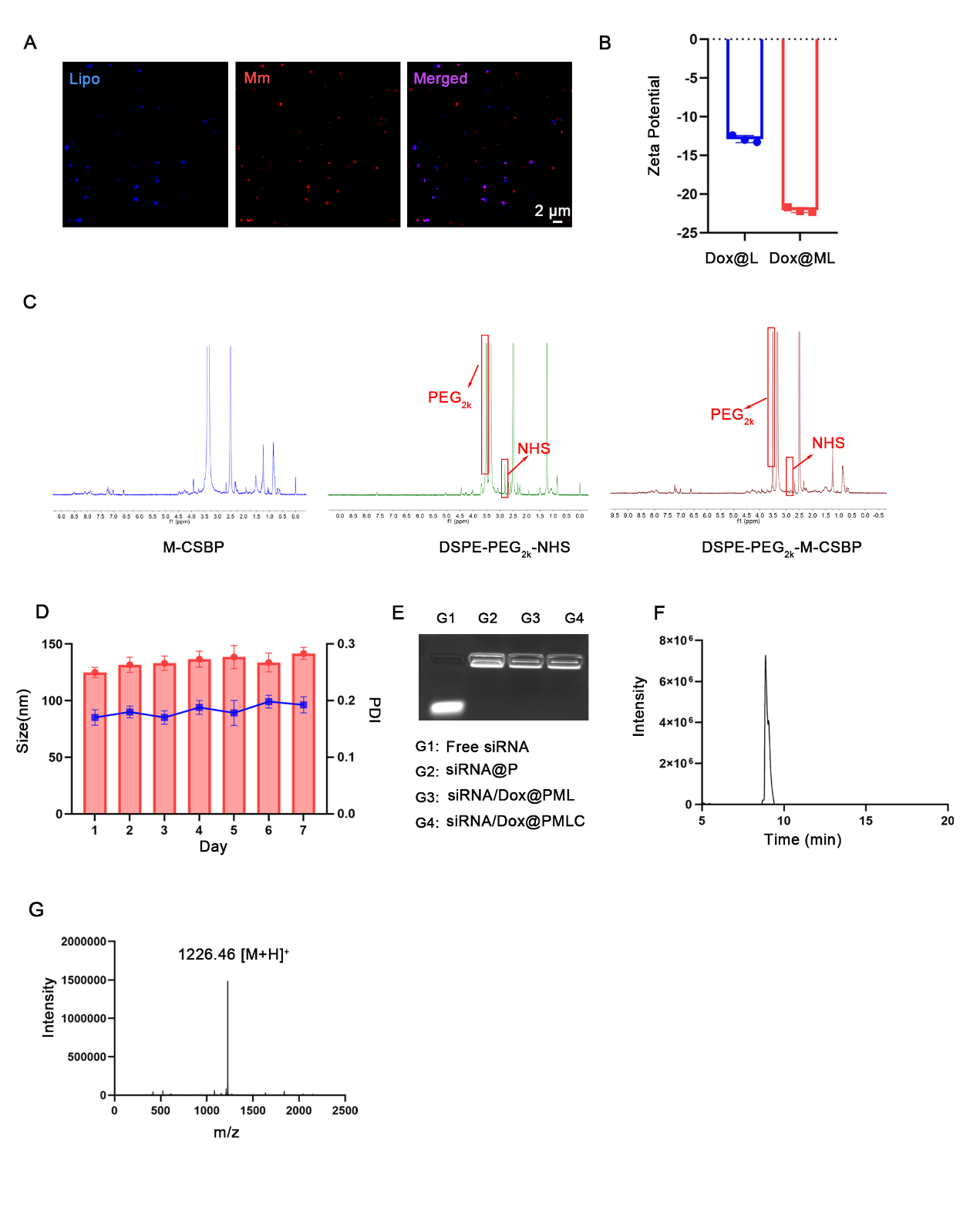


**Figure S2** (A) Confocal microscopy images of Lipo and Mm co-localization. (Lipo labeled with DiD, blue; Mm labeled with DiR, red; scale bar: 2 μm) (B) Zeta potentials of Dox@L and Dox@ML, *n* = 3. (C) ^1^H nuclear magnetic resonance (NMR) spectra of M-CSBP, DSPE-PEG-NHS and DSPE-PEG-M-CSBP. (D) Stability of siRNA/Dox@PMLC in culture medium supplemented with FBS, *n* = 3. (E) Agarose gel electrophoresis of siXkr8@P, siXkr8/Dox@PML, siXkr8/Dox@PMLC. (F-G) The liquid phase absorption peaks and the corresponding mass spectra of siXkr8/Dox@PMLC after treatment with MMP-2.


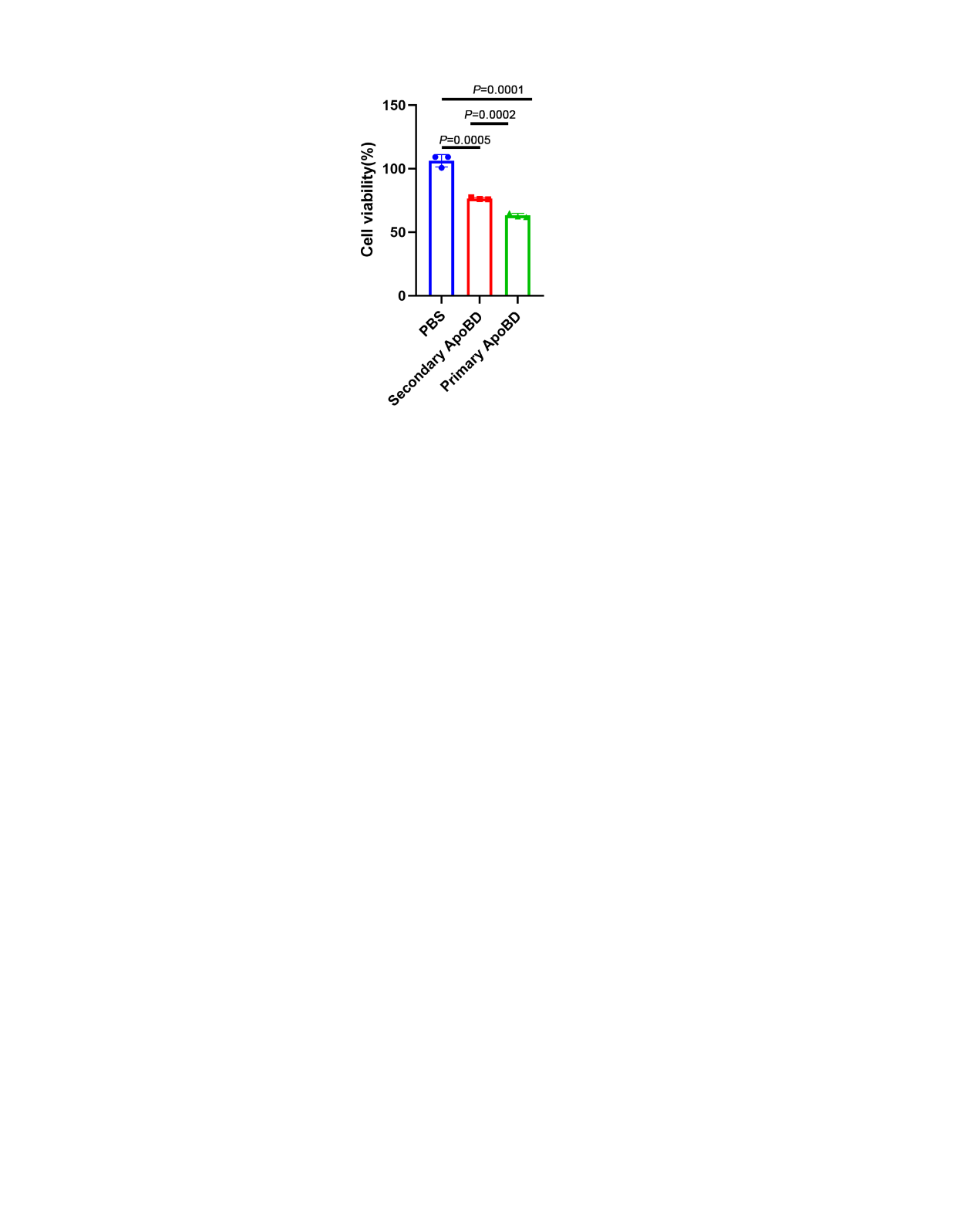


**Figure S3.** Apoptotic bodies exhibit toxicity toward CSCs. (*n* = 3, **P* < 0.05，***P* < 0.01，****P* < 0.001 )


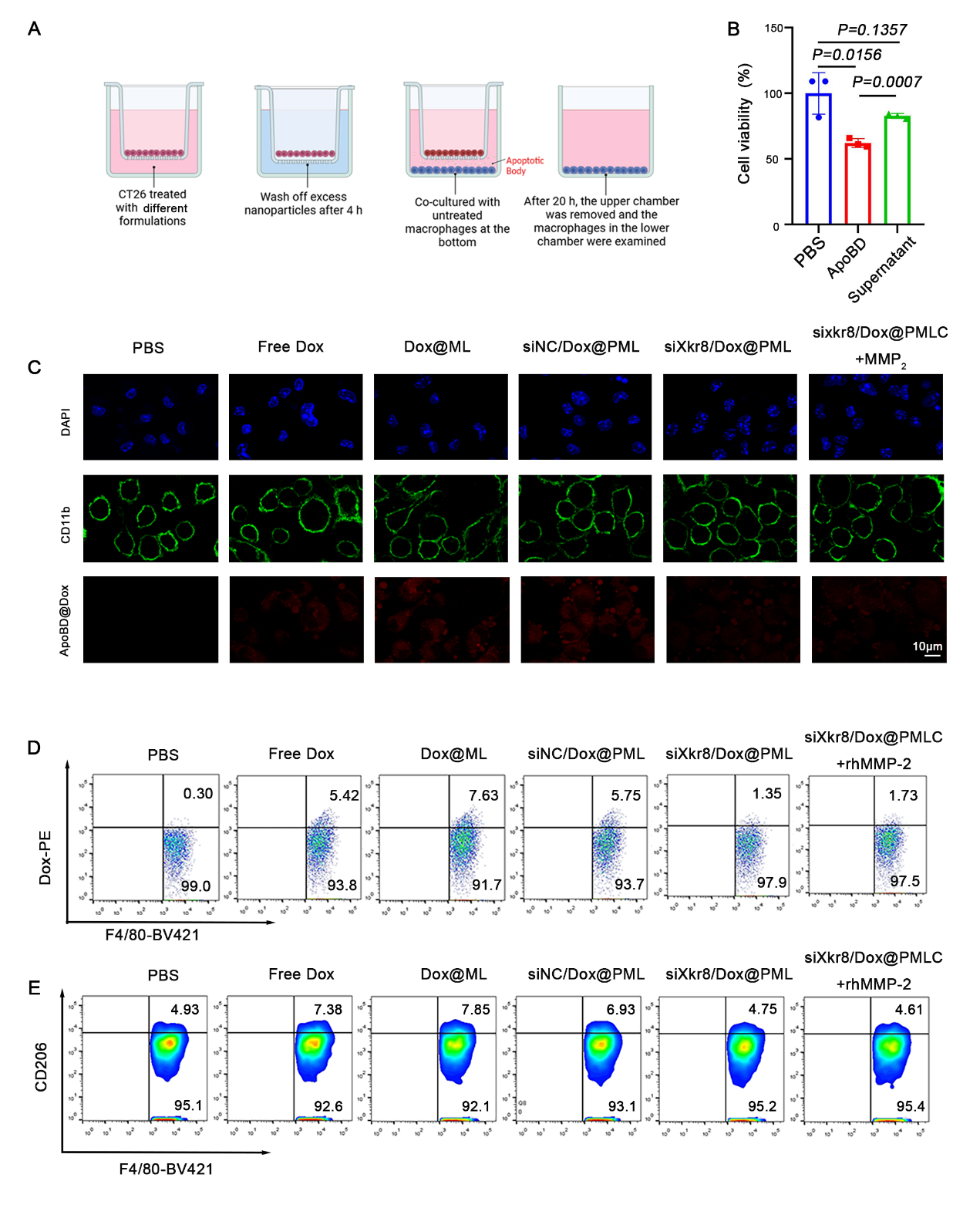


**Figure S4**. (A) Schematic diagram of Transwell setup. CT26 cells were initially co-cultured with various nanoparticles in the upper chamber for 4 hours. Thereafter, nanoparticles that were not phagocytosed by CT26 cells were removed from the upper chamber, and the incubation was continued for an additional 20 h. During this period, the produced Dox@ApoBD permeated through the microporous filter membrane into the lower chamber, which was pre-seeded with BMDM. (B) Schematic diagram of the co-culture of functionalized ApoBD with CT26 cells and BMDM. (C) Representative flow cytometry plot of Dox@ApoBD phagocytosis by BMDM. (D) Representative flow cytometry plot of M2 phenotype macrophages.

**
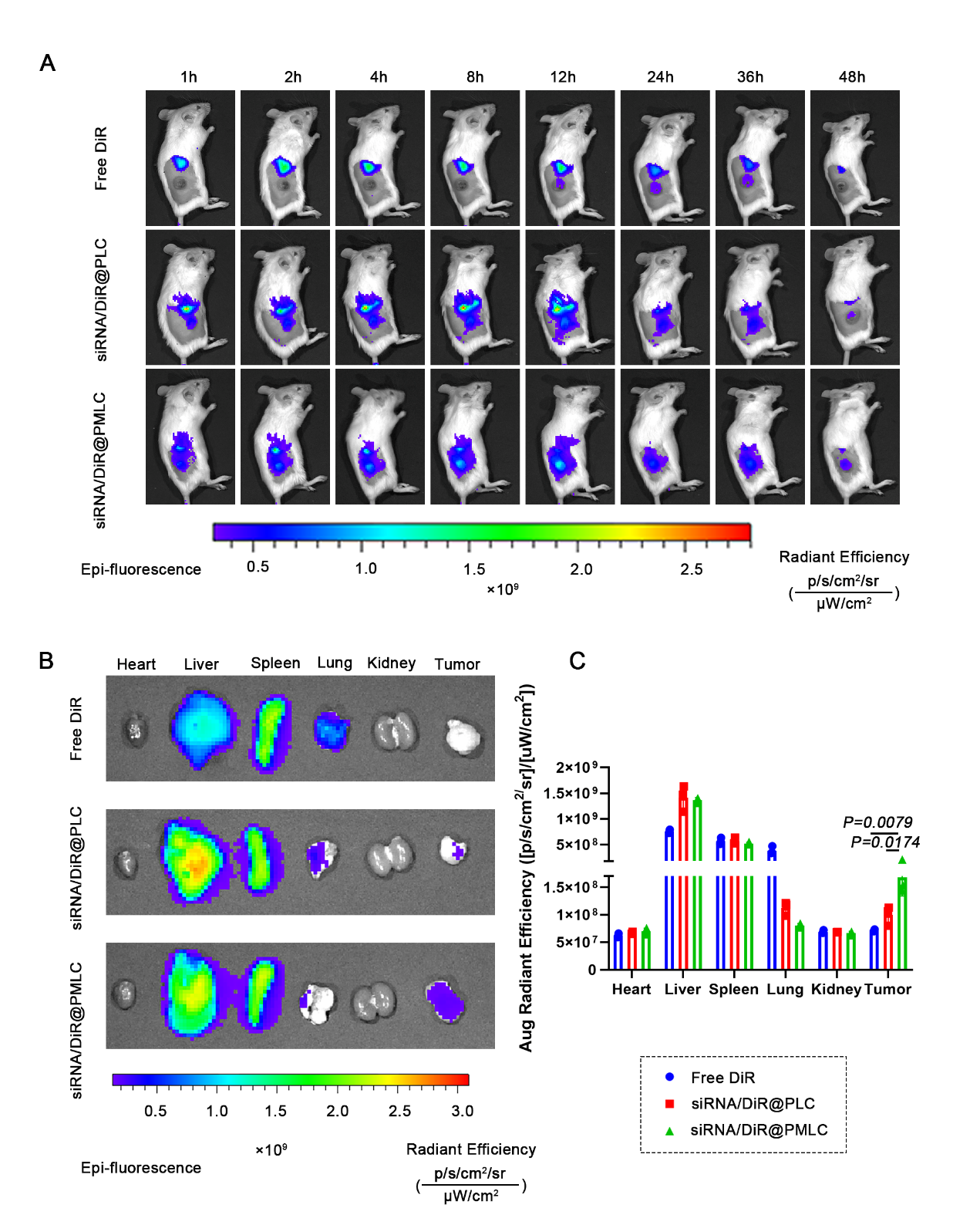
**

**Figure S5. *In vivo* distribution of nanoparticles.** (A) Fluorescence imaging of mice at 1, 2, 4, 8, 12, 24 ,36 and 48 h after injection. (B) Fluorescence images of mouse tumors and major organs. (C) Quantitative analysis of fluorescence intensity of isolated images. (*n* = 3, **P* < 0.05, ***P* < 0.01, ****P* < 0.001)


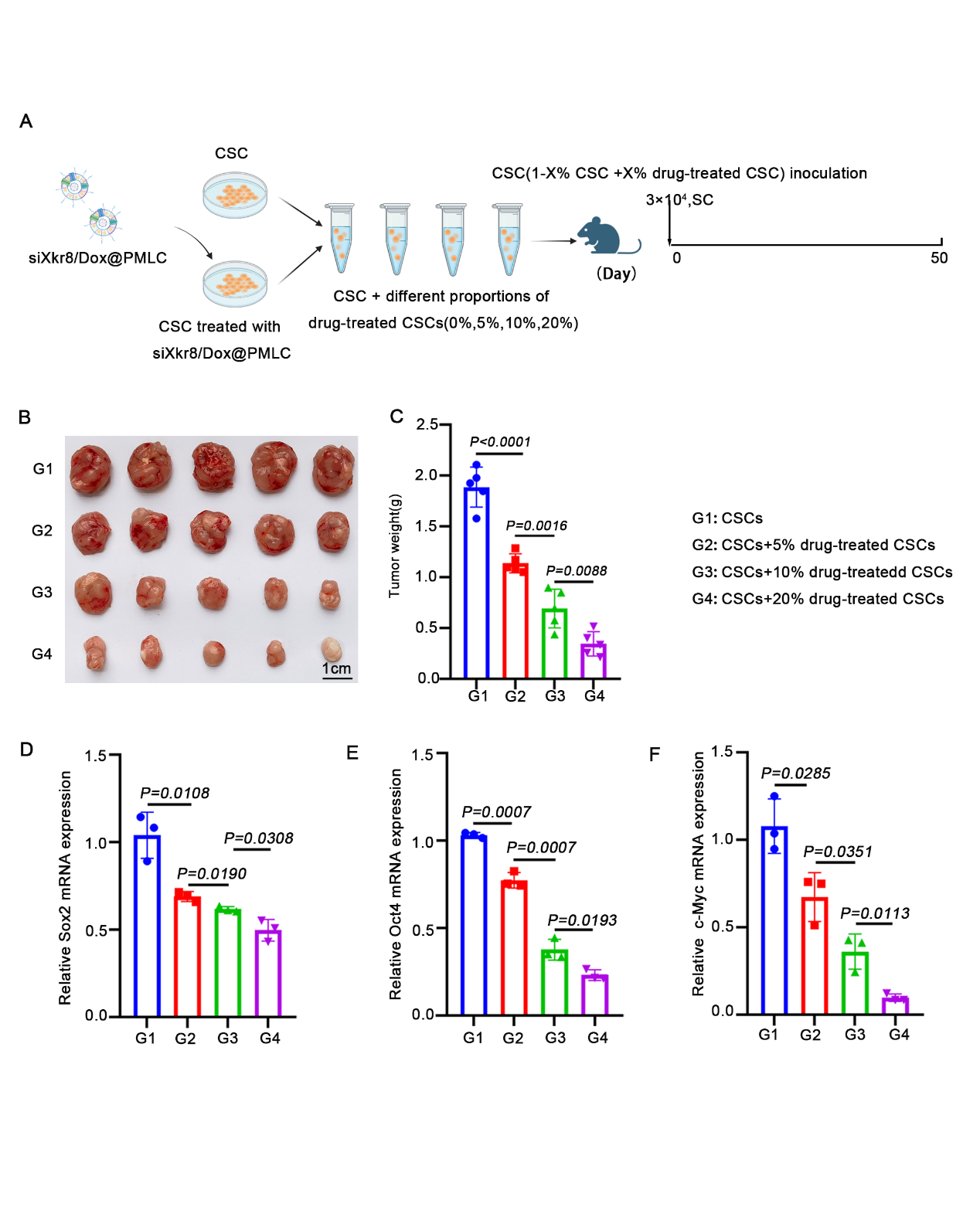


**Figure S6 siXkr8/Dox@PMLC treatment significantly inhibits the tumorigenicity of CSCs *in vivo*.** (A) Schematic illustrstion of tumorigenicity of tumor stem cells. (B) *In vitro* tumor images of different treatment groups, *n* = 5. (C) Tumor weights of mice in different treatment groups, *n* = 5. (D-F) Expression of relevant stemness genes in tumor tissues, *n* = 3.


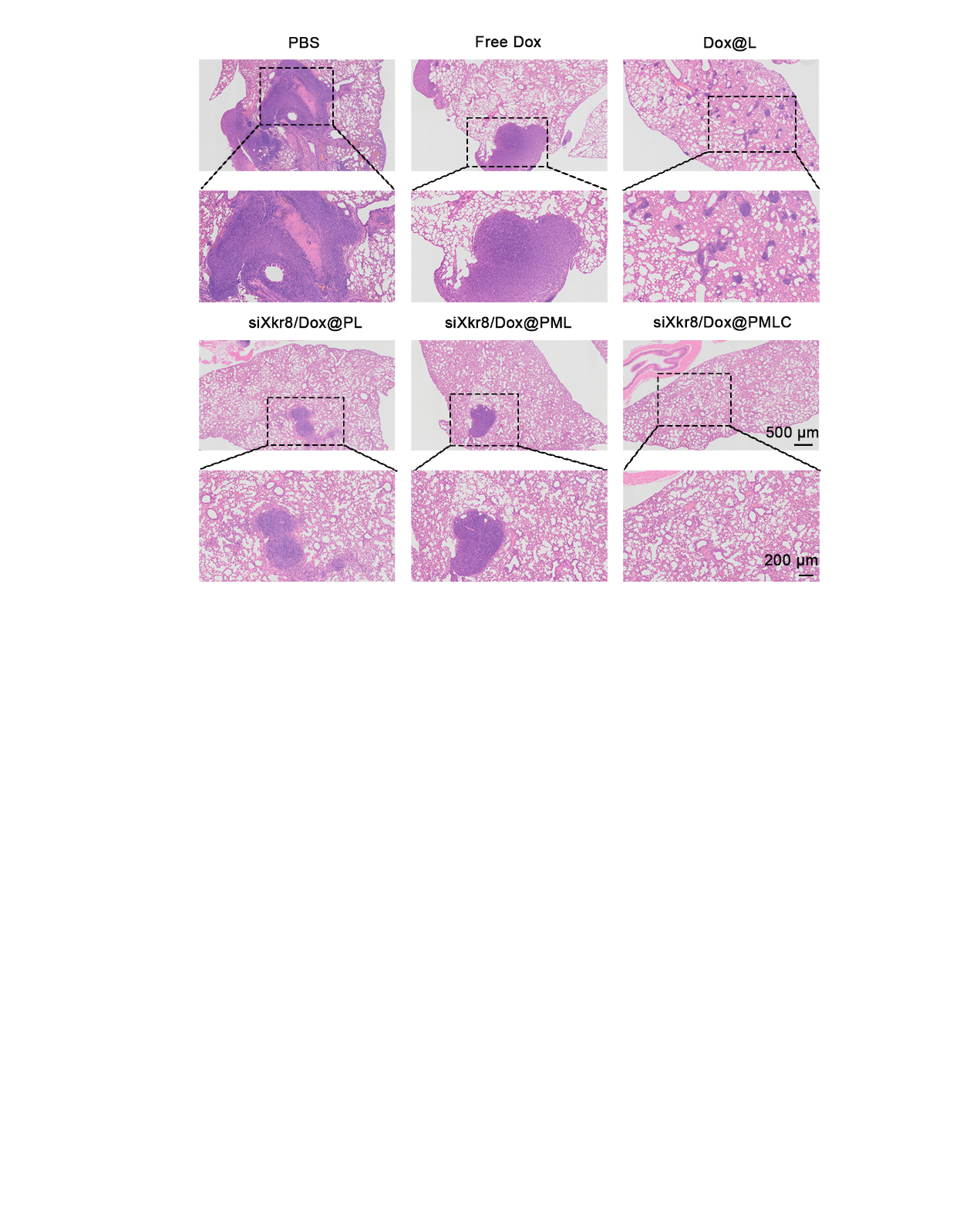


**Figure S7** Representative H&E staining of lung tissue metastases.

**
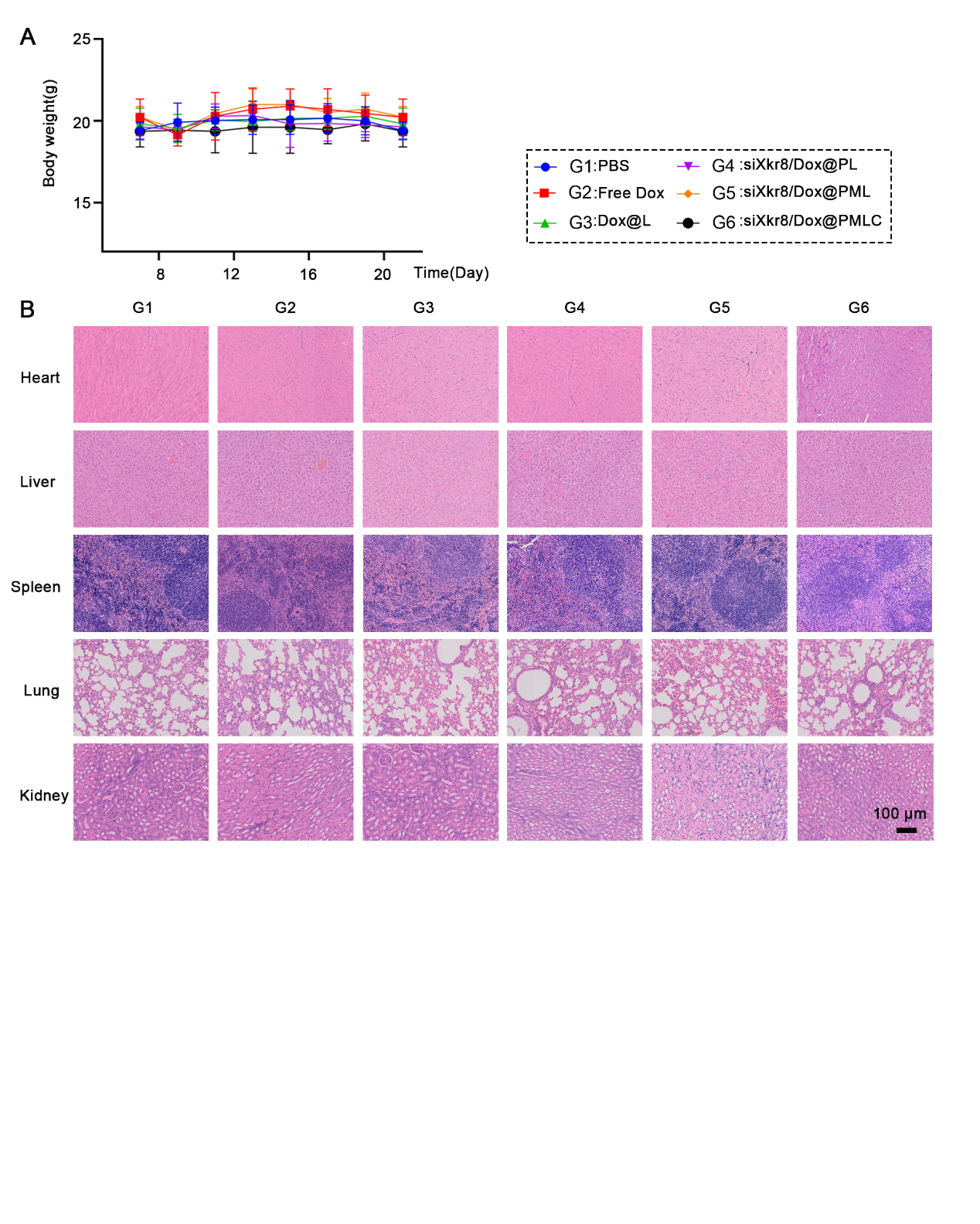
**

**Figure S8. Nanoparticle biocompatibility assessment.** (A) Change in body weight of mice during treatment. (B) H&E staining of heart, liver, spleen, lungs and kidneys in the mice of different groups. Scale bar: 100 μm.

**
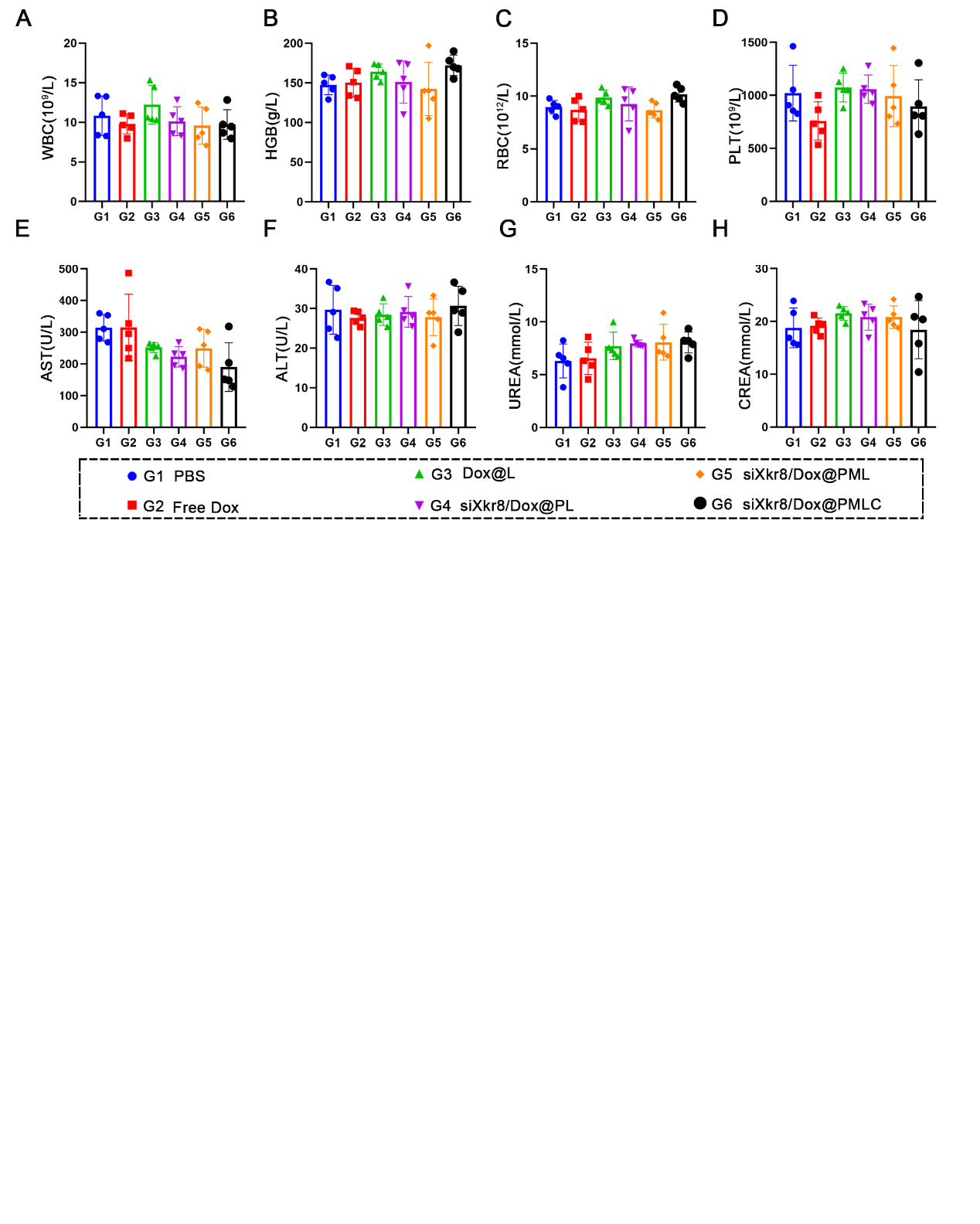
**

**Figure S9.** Blood physiology and biochemistry index of mice injected with indicated drugs, *n* = 5.

**Table S1** siRNA sequences

| **Name** | **Sense** (5’-3’) | **Antisense** (5’-3’) |
| --- | --- | --- |
| siXkr8 | CCUCUGCUAUCUACUUCCUTT | AGGAAGUAGAUAGCAGAGGTT |
| siNC | UUCUCCGAACGUGUCACGUTT | ACGUGACACGUUCGGAGAATT |

**Table S2 Primers sequences for real time qRT-PCR**

| **name** | **sequences** |
| --- | --- |
| Xkr8-F | CACTGGTGCTGGCAATTGTATT |
| Xkr8-R | CGCAGAGACCGATGGTAATCC |
| Sox2-F | GGAAAGGGTTCTTGCTGGGT |
| Sox2-R | ACGAAAACGGTCTTGCCAGT |
| Oct4-F | GAGCAGATAGGAACTTGCTGG |
| Oct4-R | AGGTTCTCATTGTTGTCGGCT |
| mGAPDH-F | GCATCCACTGGTGCTGCC |
| mGAPDH-R | TCATCATACTTGGCAGGTTTC |
