# Supplementary material for: In Situ Engineered “Cascade‐Amplified” Drug‐Loaded Vesicles for Enhanced Cancer Stem Cell Therapy
Source: J Extracell Vesicles. 2026 May 9;15(5):e70292. doi: 10.1002/jev2.70292 (PMC13157588; doi:10.1002/jev2.70292)
Supplement: Supplementary file 2 — Supporting Tables: jev270292‐sup‐0002‐tablesS1‐S2.docx [file JEV2-15-e70292-s007.docx]

**Table S1** siRNA sequences

| **Name** | **Sense** (5’-3’) | **Antisense** (5’-3’) |
| --- | --- | --- |
| siXkr8 | CCUCUGCUAUCUACUUCCUTT | AGGAAGUAGAUAGCAGAGGTT |
| siNC | UUCUCCGAACGUGUCACGUTT | ACGUGACACGUUCGGAGAATT |

**Table S2 Primers sequences for real time qRT-PCR**

| **name** | **sequences** |
| --- | --- |
| Xkr8-F | CACTGGTGCTGGCAATTGTATT |
| Xkr8-R | CGCAGAGACCGATGGTAATCC |
| Sox2-F | GGAAAGGGTTCTTGCTGGGT |
| Sox2-R | ACGAAAACGGTCTTGCCAGT |
| Oct4-F | GAGCAGATAGGAACTTGCTGG |
| Oct4-R | AGGTTCTCATTGTTGTCGGCT |
| mGAPDH-F | GCATCCACTGGTGCTGCC |
| mGAPDH-R | TCATCATACTTGGCAGGTTTC |
