# Supplementary figures and images for: In Situ Engineered “Cascade‐Amplified” Drug‐Loaded Vesicles for Enhanced Cancer Stem Cell Therapy
Source: J Extracell Vesicles. 2026 May 9;15(5):e70292. doi: 10.1002/jev2.70292 (PMC13157588; doi:10.1002/jev2.70292)

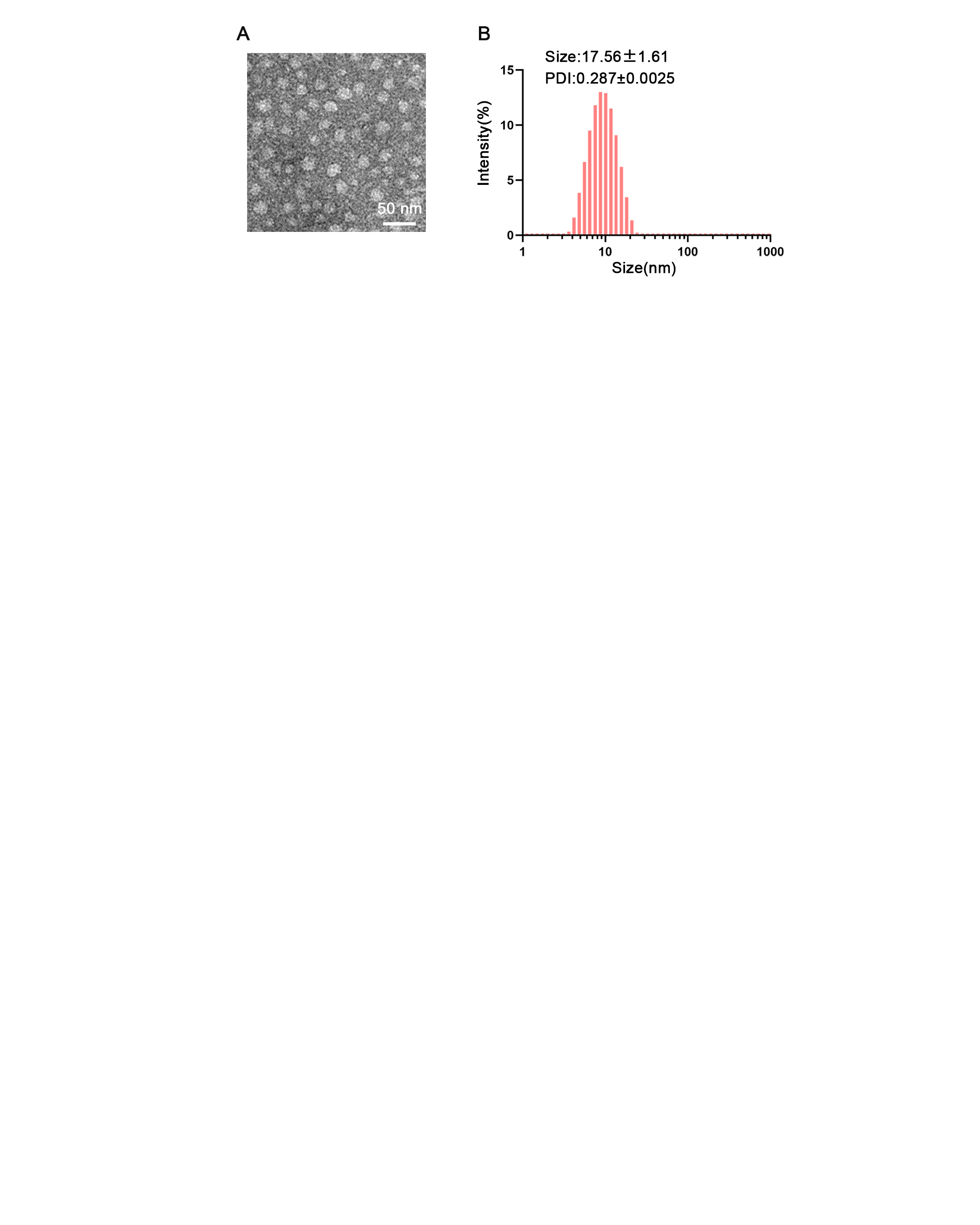

Supplement: Supplementary file 3 — Supporting Figure: jev270292‐sup‐0003‐figureS1.tif [file JEV2-15-e70292-s008.tif]

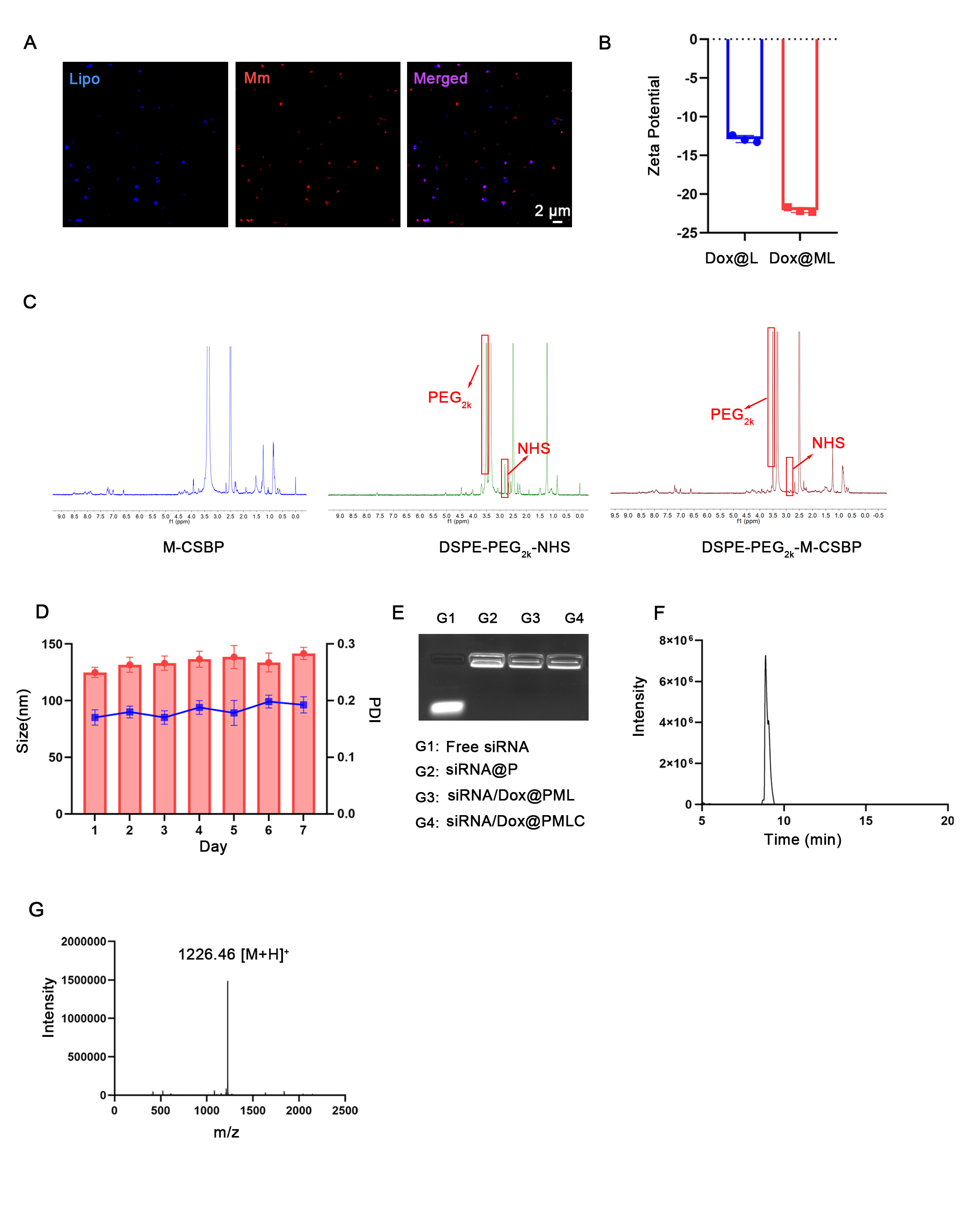

Supplement: Supplementary file 4 — Supporting Figure: jev270292‐sup‐0004‐figureS2.tif [file JEV2-15-e70292-s009.tif]

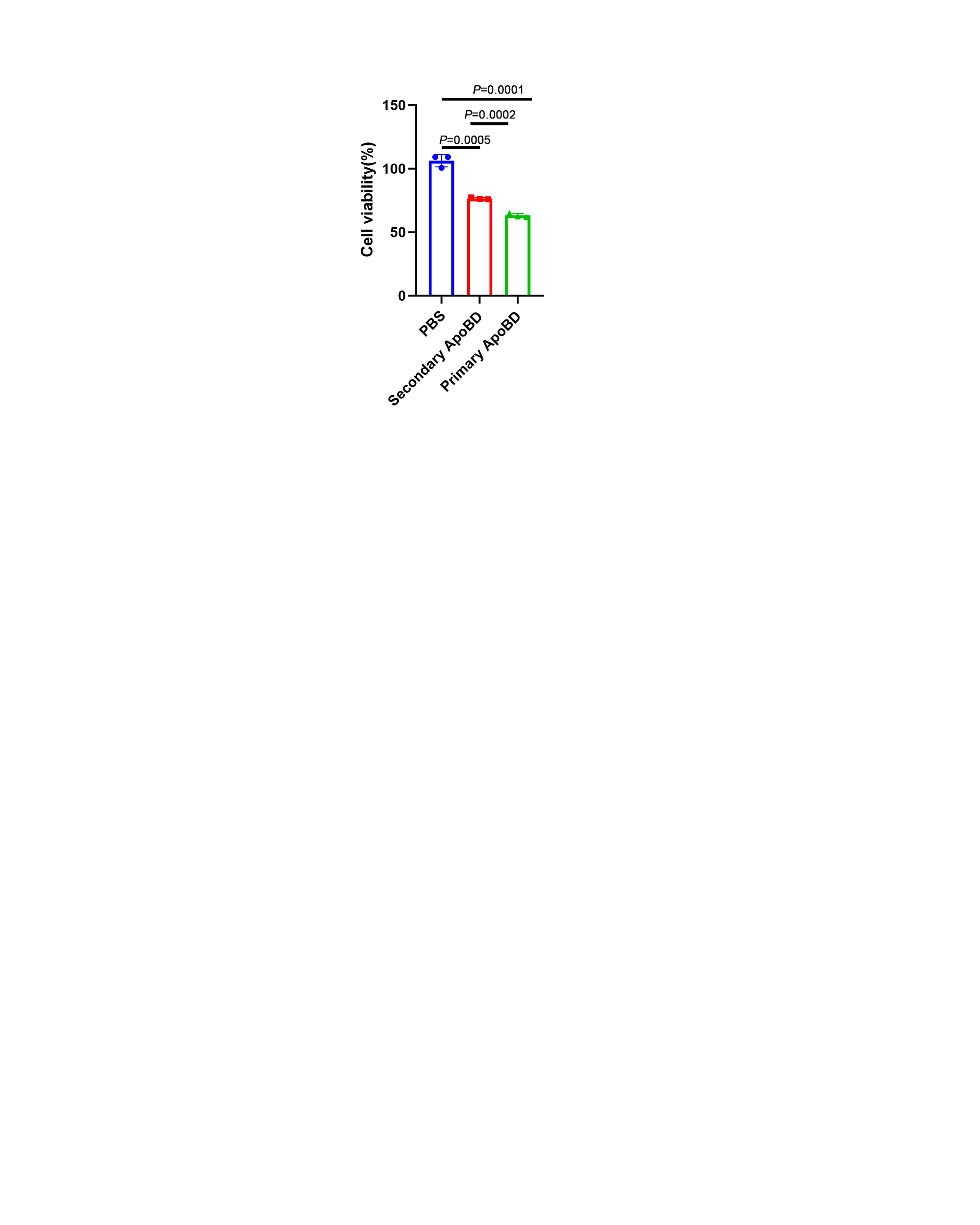

Supplement: Supplementary file 5 — Supporting Figure: jev270292‐sup‐0005‐figureS3.tif [file JEV2-15-e70292-s003.tif]

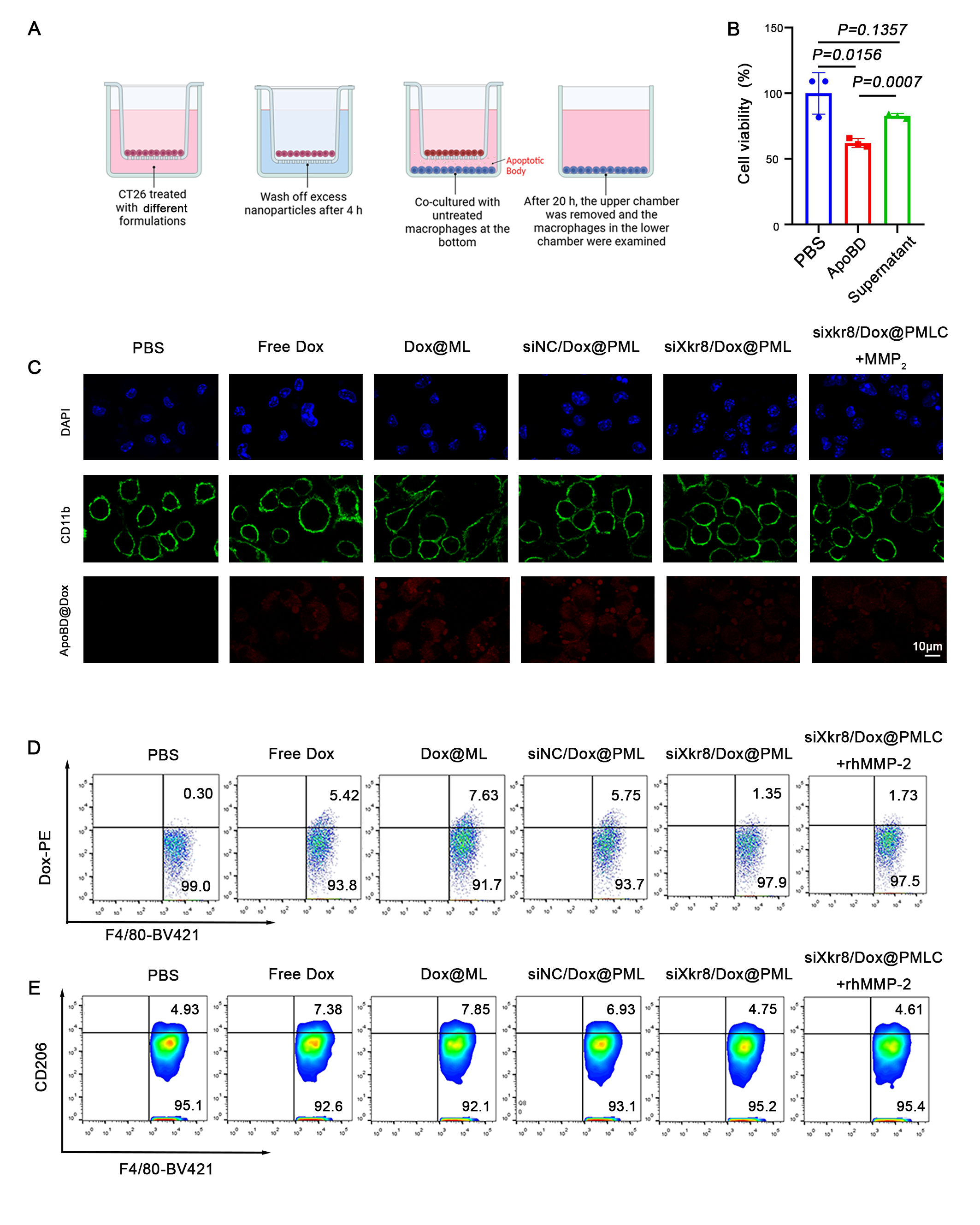

Supplement: Supplementary file 6 — Supporting Figure: jev270292‐sup‐0006‐figureS4.tif [file JEV2-15-e70292-s011.tif]

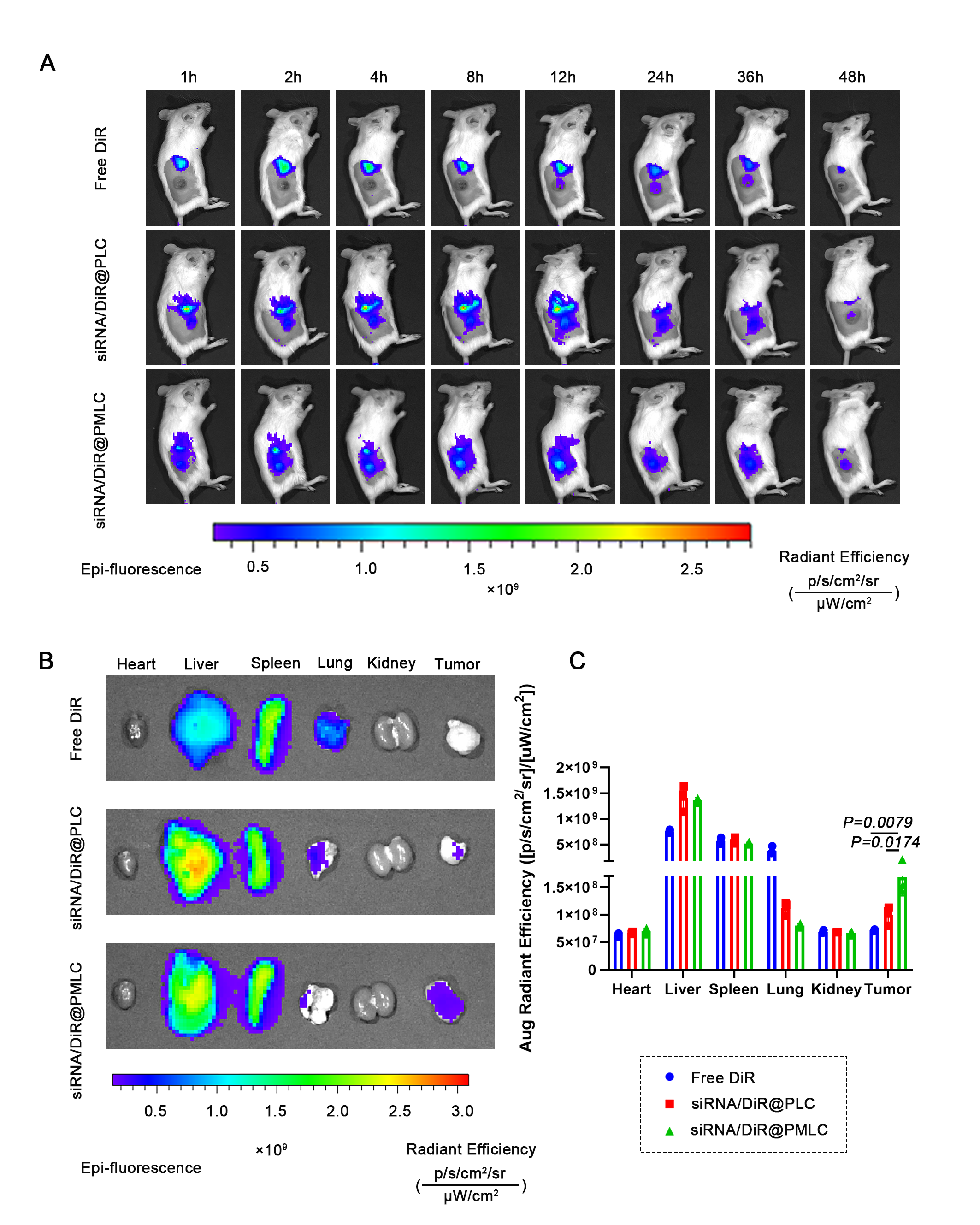

Supplement: Supplementary file 7 — Supporting Figure: jev270292‐sup‐0007‐figureS5.tif [file JEV2-15-e70292-s005.tif]

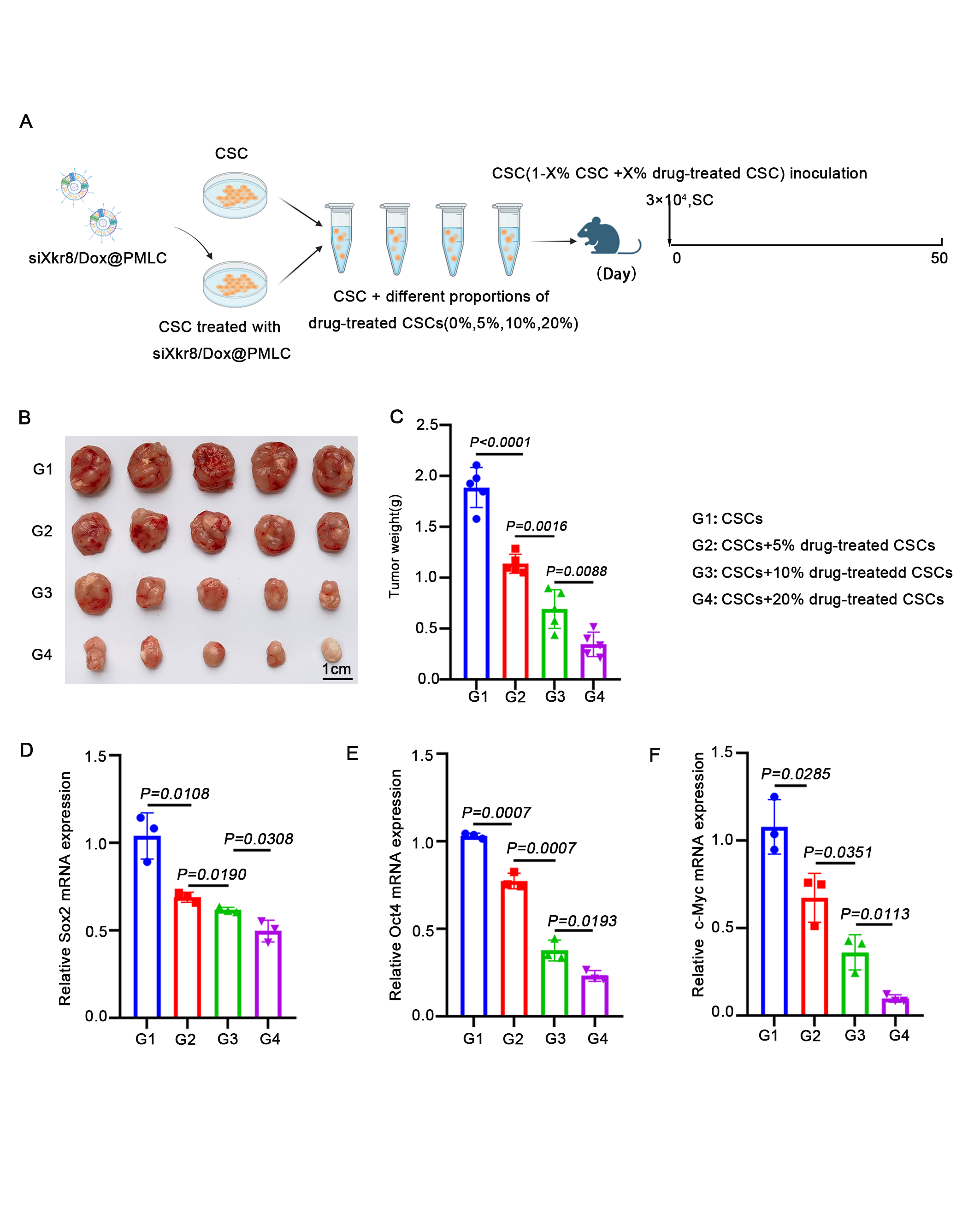

Supplement: Supplementary file 8 — Supporting Figure: jev270292‐sup‐0008‐figureS6.tif [file JEV2-15-e70292-s006.tif]

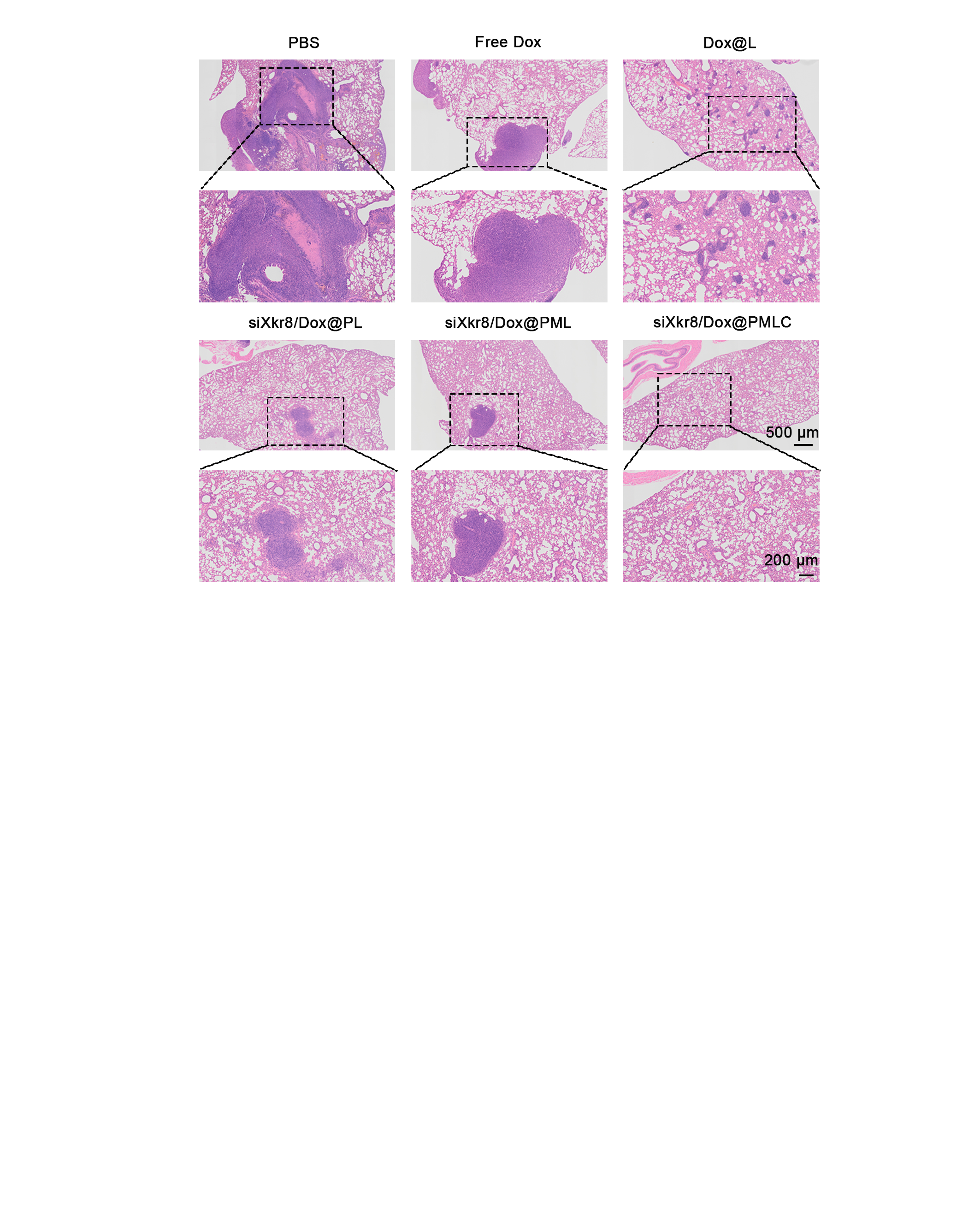

Supplement: Supplementary file 9 — Supporting Figure: jev270292‐sup‐0009‐figureS7.tif [file JEV2-15-e70292-s001.tif]

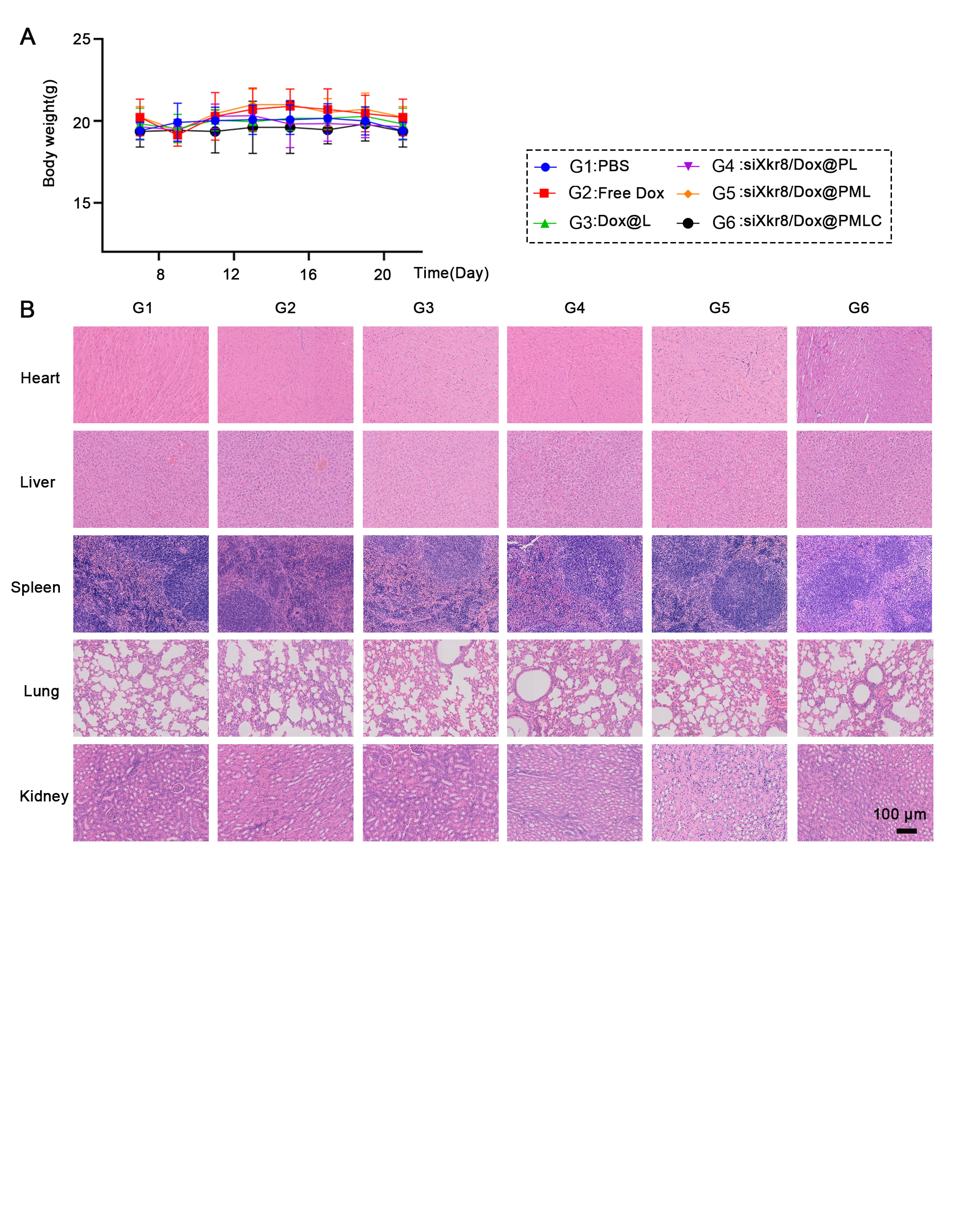

Supplement: Supplementary file 10 — Supporting Figure: jev270292‐sup‐0010‐figureS8.tif [file JEV2-15-e70292-s010.tif]

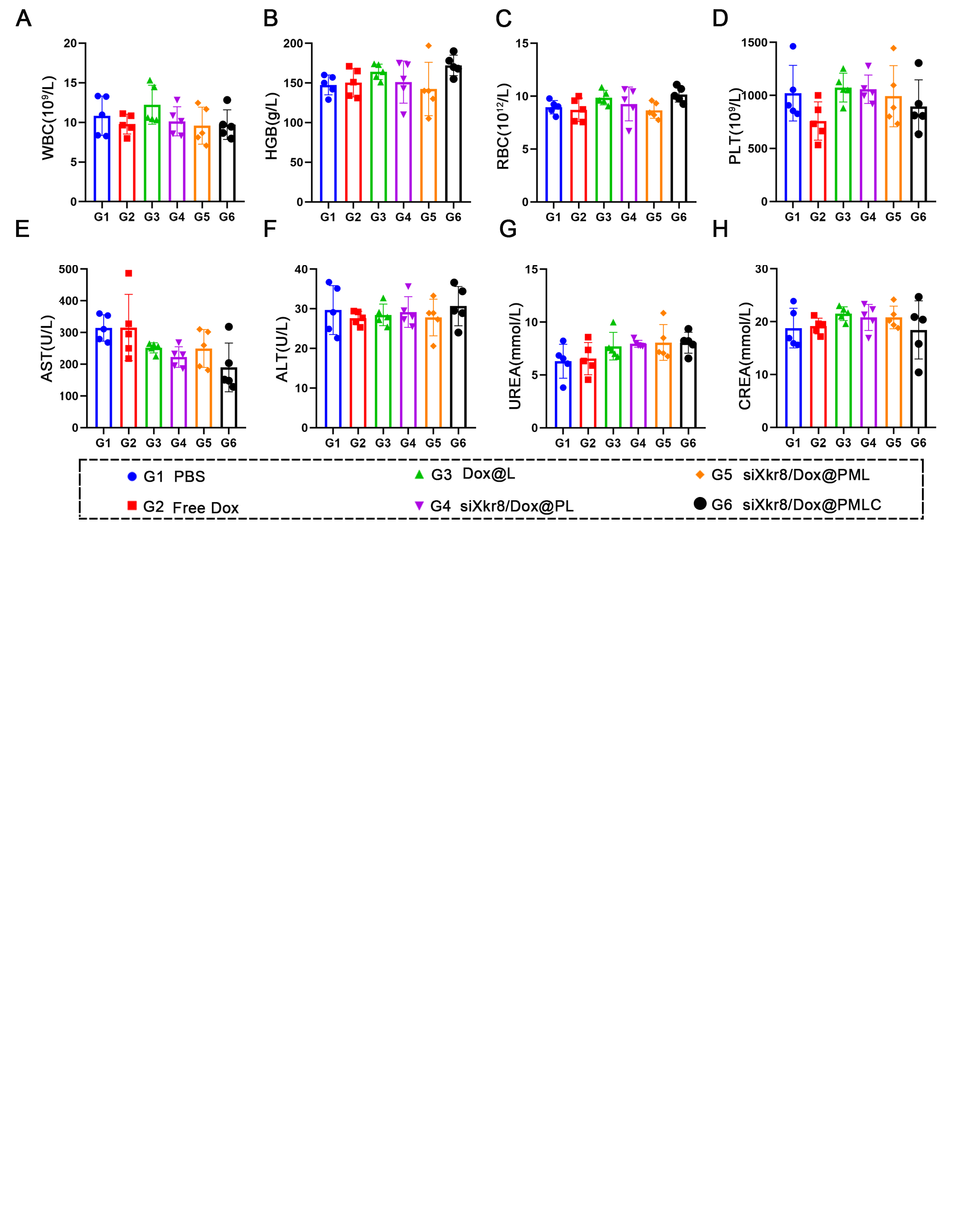

Supplement: Supplementary file 11 — Supporting Figure: jev270292‐sup‐0011‐figureS9.tif [file JEV2-15-e70292-s002.tif]
